# Antioxidant activity, anti-tyrosinase activity, molecular docking studies, and molecular dynamic simulation of active compounds found in nipa palm vinegar

**DOI:** 10.7717/peerj.16494

**Published:** 2023-11-24

**Authors:** Moragot Chatatikun, Aman Tedasen, Nawanwat Chainuwong Pattaranggoon, Wilawan Palachum, Sirithip Chuaijit, Amron Mudpan, Supawita Pruksaphanrat, Sasirat Sohbenalee, Kenshi Yamasaki, Wiyada Kwanhian Klangbud

**Affiliations:** 1 Department of Medical Technology, School of Allied Health Sciences, Walailak University, Thasala, Nakhon Si Thammarat, Thailand; 2 Center of Excellence Research of Melioidosis and Microorganisms, Walailak University, Thasala, Nakhon Si Thammarat, Thailand; 3 Research Excellence Center of Innovation and Health Products, Walailak University, Thasala, Nakhon Si Thammarat, Thailand; 4 Program in Bioinformatics and Computational Biology, Chulalongkorn University, Bangkok, Thailand; 5 Faculty of Medical Technology, Rangsit University, Muang Pathumthani, Pathumthani, Thailand; 6 School of Medicine, Walailak University, Thasala, Nakhon Si Thammarat, Thailand; 7 Department of Dermatology, Graduate School of Medicine, Tohoku University, Sendai, Japan

**Keywords:** Nypa fruticans Wurmb, Antioxidant, Molecular docking, Nipa palm vinegar, Molecular dynamic, Tyrosinase inhibitor

## Abstract

Tyrosinase is a key enzyme in melanogenesis and its inhibitors have become increasingly because of their potential activity as hypopigmenting agents which have less side effects. Nipa palm vinegar is an aqueous product that is normally used as a food supplement. The aim of this study was to study the determination of antioxidant activity and tyrosinase inhibitory activities of aqueous extract of original nipa palm vinegar (AE O-NPV), nipa palm vinegar powder (NPV-P) and aqueous extract of nipa palm vinegar powder (AE NPV-P) were examined. Nipa palm vinegars were evaluated the phenolic and flavonoid content, and the active compounds which were submitted to molecular docking and molecular dynamic simulation, chemoinformatics, rule of five, skin absorption and toxicity. The highest phenolic and flavonoid contents in the AE O-NPV were 2.36 ± 0.23 mg gallic acid equivalents/g extract and 5.11 ± 0.59 mg quercetin equivalents/g, and the highest ABTS radical cation scavenging activity was also found. The AE O-NPV, NPV-P and AE NPV-P showed anti-mushroom tyrosinase activity. The HPLC analysis showed that there were vanillic acid and three flavonoids (catechin, rutin and quercetin). The molecular docking study revealed that the binding of the vanillic acid and three flavonoids occurred in the active site residues (histidine and other amino acids). Moreover, the number of hydrogen bond acceptors/donors, solubility, polar surface area and bioavailability score of the vanillic acid and three flavonoids were acceptable compared to Lipinski’s Rule of Five. The molecular dynamic simulation showed that vanillic acid interacts with HIS284 through *π*–*π* stacking hydrophobic interactions and forms a metal-acceptor interaction with the copper molecule at the tyrosinase active site. All compounds revealed good skin permeability and nontoxicity. Nipa palm vinegar could be a promising source of a new ingredient for tyrosinase inhibition for cosmetics or pharmaceutical products.

## Introduction

Tyrosinase, dinuclear copper-containing metalloenzyme, is an important enzyme associated with melanin synthesis in melanosomes (Obaid et al., 2021). Catalyzed by the enzyme tyrosinase, the process of melanogenesis consists of two sequential steps. Initially, the tyrosinase enzyme catalyzes the rate-limiting step of the hydroxylation of L-tyrosine to 3,4-dihydroxyphenylalanine (L-DOPA), the oxidation of L-DOPA to DOPA-quinone and further products, and finally forming brown to black melanin (Mikami et al., 2013). The eumelanin, as the main natural biological pigment, is responsible for protecting human skin against ultraviolet radiation. Nevertheless, the excess production of melanin or a high melanin content causes hyperpigmentation disorders, such as post-inflammatory hyperpigmentation, melasma, solar lentigines and freckles (Plensdorf, Livieratos & Dada, 2017). There are many factors that can induce an increase in melanin production, such as sun exposure, hormonal influences, age and skin inflammation (Lee, 2021). Hyperpigmentation stands as a significant global health concern, necessitating further research in the synthesis of effective antihyperpigmentation drugs. Addressing issues linked with current treatments, like toxicity and drug resistance, underscores the demand for innovative solutions. Controlling the tyrosinase-dependent melanogenesis mechanism forms a basis for potential antimelanoma therapy and effective reduction of melanin synthesis, addressing skin hyperpigmentation disorders (Ferro et al., 2018; Mughal et al., 2022). The majority of skin-lightening products found in the market rely on tyrosinase inhibitors, and a number of encouraging tyrosinase inhibitors have found applications in pharmaceuticals, cosmeceuticals, and agriculture (Hassan, Shahzadi & Kloczkowski, 2023). Nonetheless, the existing tyrosinase inhibitors are hindered by toxicity and/or limited effectiveness, driving a continuous search for improved inhibitors sourced from nature, as they are anticipated to offer a safer profile devoid of adverse effects (Di Petrillo et al., 2016). Furthermore, the need for potent new tyrosinase inhibitors is growing across diverse applications in the food, cosmetics, pharmaceutical, and clinical sectors (Zolghadri et al., 2019).

Many prescription depigmenting agents are still used to treat skin pigmentation disorders. These depigmenting agents are hydroquinone, arbutin, azelaic acid, ascorbic acid and resveratrol. Generally, hydroquinone is the gold standard agent for treating pigmentary disorders, especially melasma (Liyanage et al., 2022). However, hydroquinone has many side effects, including skin irritation, allergic contact dermatitis and exogenous ochronosis (Juliano, 2022). Kojic acid, another established tyrosinase inhibitor, demonstrates clinical efficacy and has the potential to serve as a therapeutic agent for facial aesthetic procedures and various significant dermatological conditions associated with melanin hyperpigmentation in human skin (Obaid et al., 2021). Derived from secondary fungal metabolites, kojic acid functions by chelating with the copper ions within the active site of tyrosinase, which is the primary functional group responsible for inhibiting tyrosinase activity (Peng et al., 2022). The three-dimensional structure of tyrosinase molecule reveals an active site that contains binuclear copper ions (CuA and CuB) chelated with six histidine (HIS) residues. CuA interacts with HIS61, HIS85 and HIS94, while CuB interacts with HIS259, HIS263 and HIS296. Another active cavity also contains several amino acids near the cavity entrance, which can induce substrates to the active site of the tyrosinase (Song et al., 2022). However, kojic acid, the most widely used skin-whitening agent, cause possible side effects being dermatitis, sensitization and erythema (Gillbro & Olsson, 2011), and possible tumor promotion and weak carcinogenicity in animal model (Burnett et al., 2010). Therefore, it is crucial to discover novel tyrosinase inhibitors that can effectively mitigate these unwanted effects, making it valuable and advantageous for both the pharmaceutical and cosmetic industries to focus on targeting active sites and the amino acid residues around the core. Numerous safer, more efficient, and economical tyrosinase inhibitors have been identified and documented, including examples like the structure-based design and synthesis of 2-phenylchromone derivatives (Ashraf et al., 2021), 3-hydroxyflavone derivatives (Ashraf et al., 2020), synthetic aurone derivatives (Alshaye et al., 2023), and 2-arylchromone-4-thione derivatives (Mughal et al., 2022). These studies utilized a multi-step chemical approach, involving the synthesis of new compounds. *In vitro* screening was conducted to assess their tyrosinase inhibitory activity, alongside molecular modeling simulations aimed at uncovering their binding interactions with the active sites of the tyrosinase enzyme. Furthermore, the identification of natural products, particularly flavonoids and phenolic compounds, as tyrosinase inhibitors underscores the potential of harnessing nature’s resources for novel therapeutic interventions (Lee, Baek & Nam, 2016). Phenolic compounds are natural bioactive molecules and a main class of secondary metabolites in plants, showing a broad range of biological activities (Zhang et al., 2022). These natural compounds often possess intricate molecular structures that align with the active sites of tyrosinase, allowing them to effectively impede the melanin synthesis process. Consequently, these challenges have captured the attention of numerous researchers who are motivated to discover natural tyrosinase inhibitors that possess qualities of safety, effectiveness, and strong efficacy.

*Nypa fruticans* Wurmb., known as nipa palm or mangrove palm, belongs to the family Arecaceae. The products of nipa palm are sugar syrup, vinegar, roof thatching, wall partitioning, cigarette paper and ferment sap (Hossain, 2015). Nipa palm vinegar showed an immunomodulatory effect (Laklaeng & Kwanhian, 2020), antidiabetic effect (Yusoff et al., 2015a), antilipidemic activity (Chatatikun & Kwanhian, 2020), antioxidants, anti-inflammatory and antimicrobial activities (Senghoi & Klangbud, 2021). Moreover, the previously studied long-term preserving the quality of NPV by producing spray drying NPV powder was developed (Palachum, Klangbud & Chisti, 2022). The presence of several bioactive compounds has been identified in Nipa palm vinegar, which includes gallic acid, isoquercetin, quercetin, catechin, and rutin (Chatatikun & Kwanhian, 2020). Gallic acid, also known as 3,4,5-trihydroxy benzoic acid, is a phenolic compound that is commonly found in plants. Gallic acid is categorized as a phenol group of antioxidants with several pharmacological effects such as anti-inflammatory, antioxidant, anti-carcinogenic, anti-bacterial, anti-fungal, anti-tyrosinase, and photoprotective activities (Kahkeshani et al., 2019). Isoquercetin, also known as quercetin-3-O-glucoside, is a significant glycosidic compound found in quercetin that has been found to possess various benefits including antioxidant properties, inhibition of key enzymes associated with hyperpigmentation, skin aging, diabetes, inflammation, and neurodegenerative diseases (El Maaiden et al., 2023). Extensive research has been conducted on the potential benefits of quercetin, which include antidiabetic, antibacterial, anti-inflammatory, anti-Alzheimer’s, anti-arthritic, antioxidant, cardiovascular, wound-healing, and anticancer properties (Shabir et al., 2022). Rutin, also known as 3′, 4′,5,7-tetrahydroxy-flavone-3-rutinoside, is a flavonol glycoside that possesses various pharmacological properties, such as antimicrobial, anti-inflammatory, anticancer, and antidiabetic effects (Gullón et al., 2017). However, the anti-tyrosinase activity of nipa palm vinegar was yet unknown. Thus, we aimed to identify phenolics and flavonoids of nipa palm vinegar, using HPLC, antioxidant properties, and anti-tyrosinase activity. We also performed a molecular docking study and molecular dynamic simulation, which can reveal the binding activity of tyrosinase and active compounds. Moreover, we conducted chemoinformatics, rule of five (RO5), skin absorption and toxicity assessment. Thus, a combination of biological *in vitro* studies and bioinformatics simulation describe the functional mechanisms of the active compounds from nipa palm vinegar.

## Materials & Methods

### Chemical and solvents

The Folin–Ciocalteu reagent; gallic acid; aluminum trichloride; quercetin; 3,4-dihydroxy phenylalanine; mushroom tyrosinase (T3824); kojic acid; ascorbic acid; 2,2-diphenyl-1-picrylhydrazyl (DPPH); 2,2′-azino-bis (3-ethylbenzthaioline-6-sulphonic acid); and dimethyl sulfoxide were supplied by Sigma-Aldrich (St. Louis, MO, USA). The ethyl acetate; sodium dihydrogen phosphate; sodium dihydrogen phosphate; formic acid; methanol; and ethanol were purchased from Merck (Darmstadt, Germany).

### Plant materials

The *Nypa fruticans* Wurmb (*i.e.,* nipa palm) vinegar was collected from the Khanab Nak, Subdistrict of Pak Phanang, Nakhon Si Thammarat, Thailand. The voucher specimen (01518) was deposited at the Botanic Garden, Walailak University, Nakhon Si Thammarat, Thailand.

### Plant preparation and extraction

The nipa palm vinegar (NPV) was extracted using liquid–liquid extraction as previously described by Yusoff et al. (2015a) with some modifications. Approximately 500 mL of the original NPV was prepared by fermentation of the nipa palm, as shown in our previous study (Chatatikun & Kwanhian, 2020). The original NPV was concentrated to 250 mL by a vacuum rotary evaporator at 37° C. After, the concentrated NPV was extracted with ethyl acetate solvent at a ratio of 1:1 (v/v) in a separation funnel. The upper layer of the ethyl acetate was further separated, and the aqueous layer was collected. The partitioning step of the liquid–liquid extraction between the ethyl acetate and the aqueous NPV were repeated approximately three times. Then, the aqueous layer was collected, mixed, and concentrated using a rotary evaporator at 60 ° C to remove the organic solvent; prefrozen at −20° C for 15 min; frozen at −80° C for 1 h; and lyophilized using a lyophilizer to yield an aqueous extract of the original NPV (AE O-NPV).

To produce the nipa palm vinegar powder (NPV-P), fresh nipa palm vinegar was dried using a spray drier (Palachum, Klangbud & Chisti, 2022). To obtain an NPV powder extract, 50 g of the NPV powder was dissolved in 100 mL of distilled water and then mixed with ethyl acetate solvent at a ratio of 1:1 in a separation funnel. The liquid–liquid step between the ethyl acetate and aqueous NPV was performed again three times. As well as the AE O-NPV, the aqueous layer of the NPV powder was collected, pooled and concentrated using a rotary evaporator at 60° C; prefrozen at −20° C for 15 min; frozen at −80° C for 1 h; and dried using a lyophilizer to obtain an aqueous extract of the NPV powder (AE NPV-P). Both the AE O-NPV and AE NPV-P were stored at 4° C before use.

### Determination of the extraction yields

The extraction yields of the original NPV extract and NPV powder extract were calculated using the equation: yield of extraction (%) = (extraction weight (g) ×100)/dry extract (g).

### Antioxidant properties of nipa palm vinegar

The total polyphenol content of each sample was determined according to a previous protocol defined by Kooltheat et al. (2021). Briefly, AE O-NPV, NPV-P and AE NPV-P were dissolved in dimethylsulfoxide at 1 mg/mL. Then, 60 µL of sample was mixed with 60 µL of Folin–Ciocalteu reagent and 60 µL of 0.1 M sodium carbonate solution. The reaction mixture was incubated in the dark for 60 min. The absorbance of the sample was measured at a 750 nm wavelength *via* a microplate reader (Multiskan; Thermo Fisher Scientific, Cleveland, OH, USA). Three analytical repetitions were conducted for each sample. The standard solutions were prepared from the different concentrations of gallic acid. The results were expressed in mg of gallic acid equivalents per gram of dry extract or powder (mg GAE/g dry extract or powder).

The total flavonoid content in the samples was quantified using an aluminum chloride colorimetric assay (Aremu et al., 2019). The samples were prepared at a concentration of 1 mg/mL. After, 100 µL of the sample solution was mixed with 100 µL of 2% aluminum trichloride (AlCl_3_). Following incubation for 15 min, the absorbance of the reaction mixture was measured at 435 nm against a sample blank. A calibration curve was drawn with different concentrations of quercetin. The average of three readings was used and expressed as mg of quercetin equivalents per g of dry extract or powder (mg QE/g dry extract or powder).

A DPPH•scavenging activity assay was conducted using a method previously described by Kooltheat et al. (2021), with slight modification (Kooltheat et al., 2021). The DPPH stock solution, at concentration of 0.06 M, was diluted with absolute ethanol to obtain an absorbance of approximately 0.7 ± 0.02 at 517 nm. Different concentrations of the sample were prepared in the absolute methanol. A 40 µL of the sample solution was mixed with 60 µL of the DPPH solution in a 96-well plate and incubated in a dark condition at room temperature for 30 min. The reaction mixture was measured at 517 nm. The DPPH free radical scavenging activity was calculated by the equation: DPPH free radical scavenging activity (%) = [(Abs_control_ - Abs_sample_)/Abs _control_] ×100. The assay was performed in triplicate.

ABTS radical cation scavenging assay was carried out with the slight modification of a previous method (Kooltheat et al., 2021). A total of two mL of the 7 mM ABTS stock solution was mixed with three mL of potassium persulfate (K_2_S_2_O_8_). The mixture was incubated in a dark condition for 16 h and then diluted with methanol until reaching an absorbance value of 0.7 ± 0.02 at 734 nm. For the ABTS assay, 20 µL of each sample in methanol was mixed with 180 µL of ABTS ^•+^ solution in a 96-well plate and incubated in a dark condition at room temperature for 45 min. For the control, methanol was used instead of the sample. The decrease in absorbance at 734 nm was monitored. The ABTS radical scavenging activity was calculated according to the equation: [(Abs _control_ - Abs_sample_)/Abs _control_] ×100. The results were obtained from three independent determinations.

### Anti-mushroom tyrosinase activity

The anti-mushroom tyrosinase activity of each sample was performed using L-DOPA as a substrate (Pintus et al., 2015). The reaction mixture (200 µL) contained 20 µL of mushroom tyrosinase (203 unit/mL), 20 µL of sample solution and 160 µL of 2.5 mM L-DOPA in 20 mM phosphate buffer. After incubation at 37^∘^C for 30 min, the tyrosinase activity was monitored at 475 nm for dopachrome formation. Kojic acid was used as a tyrosinase inhibitor. The percentage of tyrosinase inhibition was calculated at each concentration. The results are expressed as IC_50_ (the sample concentration is expressed as 50% of the tyrosinase activity).

### Determination of phenolic acids and flavonoids of nipa palm vinegar through high-performance liquid chromatography (HPLC) analysis

An HPLC analysis was used to determine the phenolic acids and flavonoids of the selected extract that showed anti-mushroom tyrosinase activity, as described in a previous method (Klangbud et al., 2022). The selected extract was subjected to a commercial Shimadzu HPLC analysis (Shimadzu, Kyoto, Japan) at the Research Office of Thammasart University, which contained P-400 pumps and a UV 2000 detector. The chromatographic separation was carried out with a Phenomenex C18W column (4.6 × 250 mm, 5 µm). Each sample was filtrated through a 0.45 µm pore size. The mobile phases contained formic acid and methanol at a flow rate of 1.0 mL/min. The gradient elution was 0–25 min, with a linear gradient from 80% to 30% of the formic acid; 20.1–22.0 min, 90% of the methanol and re-equilibration period of 8 min, 80% of the formic acid between the individual runs. The column temperature was 38° C, and the injection volume was 10 µL. The chromatographic operations were performed at an ambient temperature. The chromatographic determinations were triplicated. Gallic acid, procatechuic acid, vanillic acid, caffeic acid, p-coumaric acid, ferulic acid and sinapic acid served as the standards for the phenolic acids. Three flavonoids containing catechin, rutin and quercetin were used as the flavonoid standards. The identification of the compounds was performed by comparing the retention time and UV absorption spectrum with those of the standards.

### Ligand and protein structure preparation

The bioactive compounds from AE O-NPV, NPV-P and AE NPV-P, including vanillic acid (PubChem CID 8468), catechin (PubChem CID 9064), quercetin (PubChem CID 5280343) and rutin (PubChem CID 5280805), were collected from PubChem the database (https://pubchem.ncbi.nlm.nih.gov/). Known tyrosinase inhibitors, such as aloin (PubChem CID 12305761), deoxyarbutin (PubChem CID 11745519), arbutin (PubChem CID 440936), kojic acid (PubChem CID 3840) and hexylresorcinol (PubChem CID 3610), were also retrieved from the PubChem database in the form of an SDF structural file. The ligand was further optimized and translated to a PDB file using Open Babel tools. The energy minimization with conjugate (steepest descendent methods) and the addition of charges for correcting the ionization was employed to prepare the ligands. Miss hydrogen atoms and polar hydrogens were added to all of the ligands at pH 7.4. The bond order, angles and topology were assigned to optimize the structure. AutoDock tools (ADT) automatically assigned the Gasteiger charges and default atom parameters. Finally, ADT was used to write the structure into the PDBQT file format. The 3D protein structures of mushroom tyrosinase (PDB ID: 2Y9X) were obtained from the RCSB Protein Data Bank (https://www.rcsb.org) in PDB format (Yu, Fan & Duan, 2019; Mughal et al., 2022). The Protein Data Bank (PDB) is a global database of 3D structural data for major proteins. The co-crystalized ligand and water molecules were removed from the protein structure. The polar hydrogens, charges, solvation parameters and fragmental volumes for the protein were assigned using the Kollman united atoms force field by ADT. Finally, the protein structures were saved in the PDBQT file format. 

### Molecular docking study

Molecular docking was performed using AutoDock version 4.2. The Lamarckian genetic method was used to run the molecular docking experiment, which was performed with AutoDock4 software. The protein structure was configured as a rigid molecule with a flexible ligand during the procedure. A grid box of 72 × 60 × 70 cubic angstroms (Å3) *and* center at x: −7.620, y: −24.771 and z: −38.576 were used to designate the docking location on the protein target, with the grid spacing at the active site of the protein structure. The default values in ADT were used for the remaining parameters. For the conformational sampling, a fifty genetic algorithm (GA) was run with a population size of 200. The optimum pose was determined to be the one with the lowest binding energy (kcal/mol). The natural ligands or drugs were compared to the interaction of the best-docked posture of the selected compound. Then, the protein–ligand interactions were analyzed and visualized using the BIOVIA Discovery Studio Visualizer software (Accelrys, San Diego, CA, USA).

### Molecular dynamics simulation

To analyze the dynamic nature of interactions between vanillic acid bioactive compounds and deoxyarbutin, molecular dynamics (MD) simulations were employed for the study. The Discovery Studio’s protein preparation technique was used to prepare tyrosinase protein structure and protonation state of any ionizable sidechain. In the tyrosinase, six histidine molecules are coordinated with two copper ions. Hence, forcefield parameters for this metal-coordinated complex were created using the Metal Center Parameter Builder (MCPB) module of Amber20 (Li & Merz, 2016). The Amber ff14SB forcefield was used for the protein. After collecting quantum mechanical (QM) optimized geometries, the generalized amber forcefield (GAFF2) was used to develop the forcefield parameters for all substrates (Rai et al., 2022). The restrained electrostatic potential (RESP) method was utilized to determine the partial atomic charges of these compounds. The ligands were positioned within the protein’s active site to produce the first configuration. The PMEMD module of the AMBER20 package was used to run the MD simulations, and the TIP3P water model and Na+ and Cl- counterions were used to neutralize each system. The 3,000 cycles of the in-vacuo energy reduction sets were performed using the SANDER module of the AMBER20 package. Later each system was heated, equilibrated, and finally transformed into an isothermal and isobaric (NPT) (1 atm at 310 K for 1000 ps). The cpptraj module was used to determine the trajectory timestep of each ligand in the pocket site and the stability of key amino acids residues to ligands during the MD simulations for 200 ns. Using 100 snapshots of the last 10 ns of the MD simulation, the solvated interaction energy (SIE) approach was used to determine the total binding free energy (ΔGbind).

### Chemoinformatics, Lipinski’s Rule of Five (RO5)and skin absorption and toxicity assessment

Recently, computationally based prediction methods for chemoinformatics and ADMET evaluations have become increasingly common in developing natural compounds. The SwissADME server (http://www.swissadme.ch/) was utilized to screen the chemoinformatics and RO5 properties of the active components from all NPVs and identify the candidate’s molecule drug-likeness and pharmacokinetic properties. The SwissADME server also used the skin absorption and bioavailability score for pharmacokinetic predictions to develop skin care products. Lastly, the pkCSM servers (https://biosig.lab.uq.edu.au/pkcsm/) predicted the toxicity tests for the active components of the AE O-NPV, NPV-P, and AE O-NPV, including skin sensitization, carcinogenicity, AMES toxicity and hepatotoxicity.

### Data analysis

All experiments were conducted in triplicate. The data are expressed as the mean and standard deviation (mean ± SD). The data were analyzed to compare the samples and the control group by one-way ANOVA followed by Dunnett’s post hoc test; a *p*-value <0.05 was regarded as significant.

## Results

### Determination of the extraction yields

As shown in Table 1, the yield of the AE O-NPV was 9.18%, which was extracted from the original NPV using the liquid–liquid partition method. The highest yield (88.75%) was found in the AE NPV-P, which used NPV powder from a spray-dried technique and was further extracted from the NPV powder by the liquid–liquid partition method. Both the AE O-NPV and AE NPV-P was obtained from the aqueous layer.

### Antioxidant properties of nipa palm vinegar

**Figure 1:**
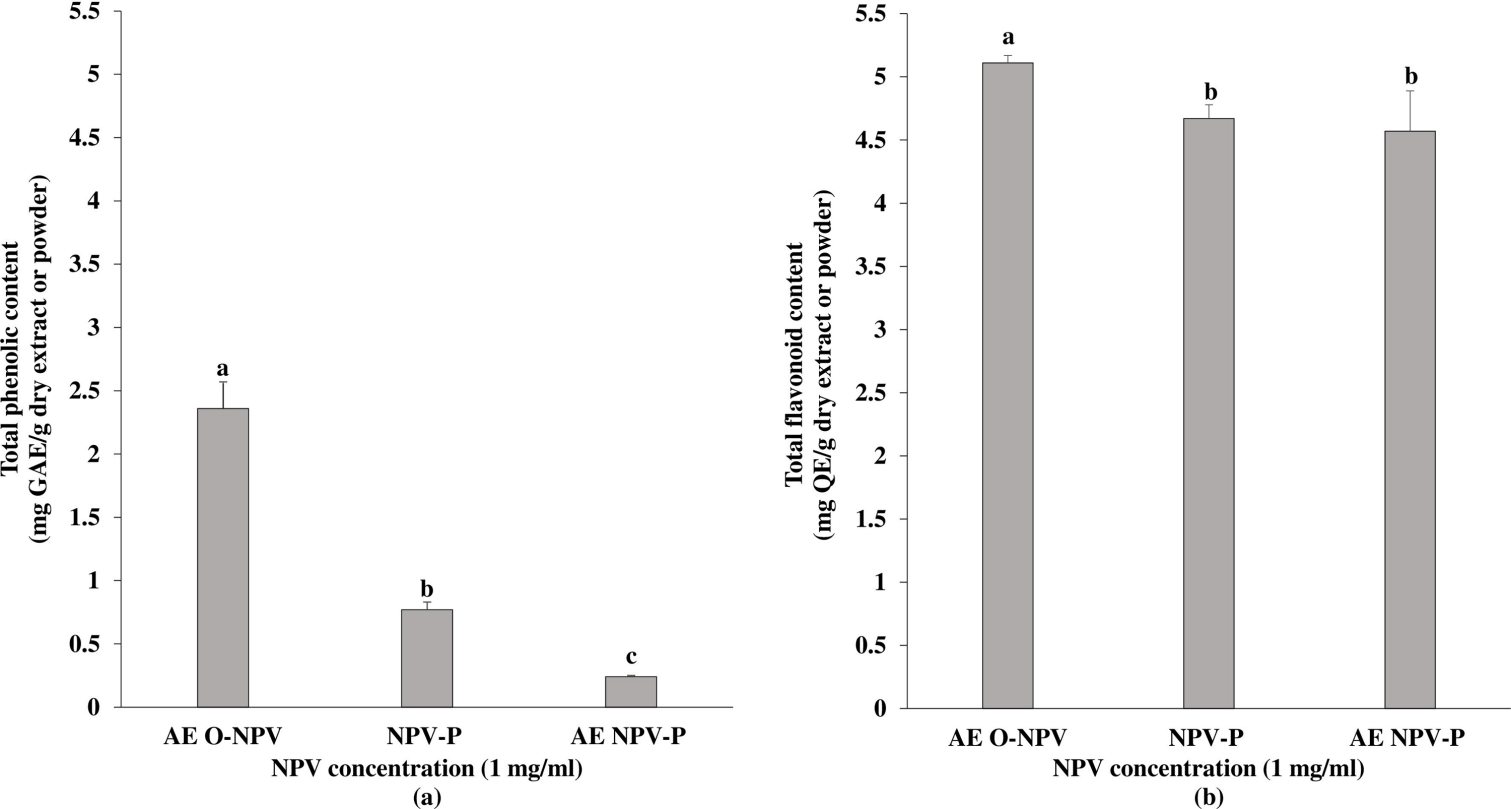
Total phenolic and flavonoid contents of the aqueous extract of nipa palm vinegar (AE O-NPV), nipa palm vinegar (NPV) and aqueous extract of nipa palm powder (AE NPV-P): (A) total phenolic content represented as mg GAE extract or powder; (B) total flavonoid content represented as mg QE extract or powder. The data are presented as the mean ± standard deviation. The bars not sharing any letters are significantly different using the Duncan multiple range test (*p* < 0.05).

The total amount of phenolic contents in the NPV samples are presented in Fig. 1. The largest amount of phenolic content (2.36 ± 0.21 mg GAE/g dry extract) was found in the AE O-NPV, whereas the phenolic contents in the NPV powder (NPV-P) and the aqueous extract of NPV powder (AE NPV-P) were 0.77 ± 0.06 mg GAE/g dry powder and 0.24 ± 0.01 mg GAE/g dry extract, respectively. The total phenolic content of the AE O-NPV was significantly higher than that of the NPV-P at *p* < 0.05. Moreover, a significantly higher phenolic content of the NPV-P was observed when compared to the AE NPV.

The total flavonoid content of the AE O-NPV, NPV-P and AE NPV-P ranged from 4.57−5.11 mg QE/g. The data shown in Fig. 1 indicate that the highest content of flavonoids was found in AE O-NPV (5.11 ± 0.06 mg QE/g dry extract), followed by NPV-P (4.67 ± 0.11 mg QE/g dry powder) and AE NPV-P (4.57 ± 0.32 mg QE/g dry extract. This result shows that the flavonoid content in the AE O-NPV were significantly higher (*p* < 0.05) than those in the NPV-P and AE NPV-P.

**Figure 2:**
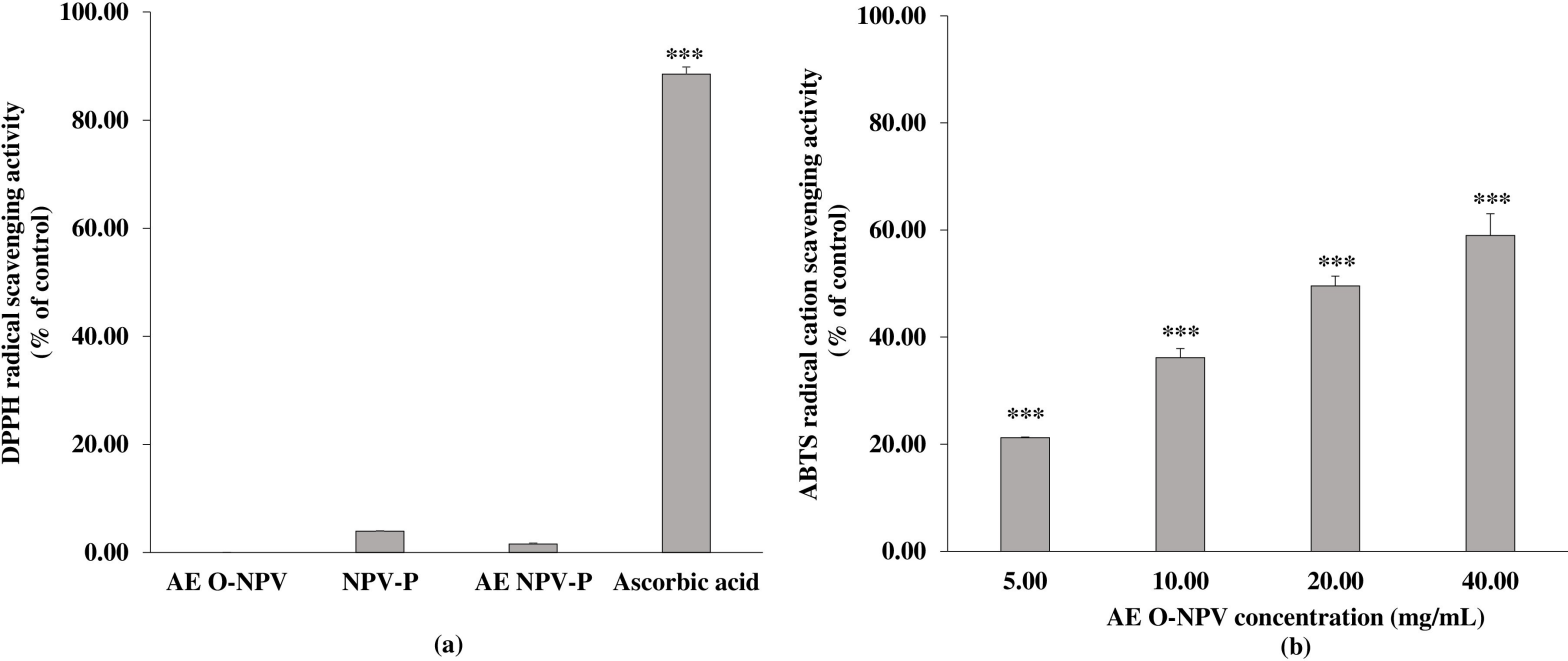
The antioxidant activity of the AE O-NPV, NPV-P and AE NPV-P at a concentration of 40.00 mg/mL was determined by (A) DPPH radical scavenging activity; (B) the ABTS radical cation scavenging activity of the AE O-NPV was determined. Each value represents the mean ± standard deviation (*n* = 3). ^∗∗∗^ *p* < 0.001, when compared AE O-NPV withcontrol.

As shown in Fig. 2A, only the NPV-P and AE NPV-P at 40.00 mg/mL exhibited DPPH scavenging activity, being 3.94 ± 0.05% and 1.58 ± 0.14% that were not significantly different from control. Moreover, the DPPH scavenging activity was not detected at 40 mg/mL of the AE O-NPV. The DPPH scavenging activity of ascorbic acid at 50 µg/mL was 88.53 ± 1.33%.

**Table 1:** Percentage of yield extract of nipa palm vinegar.

**Extract**	**%Extraction yield**
Aqueous extract of original NPV (AE O-NPV)	9.18
Aqueous extract of NPV powder (AE NPV-P)	88.75

Using another method, only the AE O-NPV exhibited concentration-dependent ABTS radical cation scavenging activities at a concentration of 5.00–40.00 mg/mL that were significantly different from control at *p* <0.001 (as shown in Fig. 2B). The SC_50_ of the AE O-NPV was 22.78 ± 1.35 mg/mL. Both the NPV-P and AE NPV-P did not exhibit ABTS free radical scavenging activity. The SC_50_ of ascorbic acid as an antioxidant agent was 41.24 ± 0.21 µg/mL.

### Anti-mushroom tyrosinase activity

**Figure 3:**
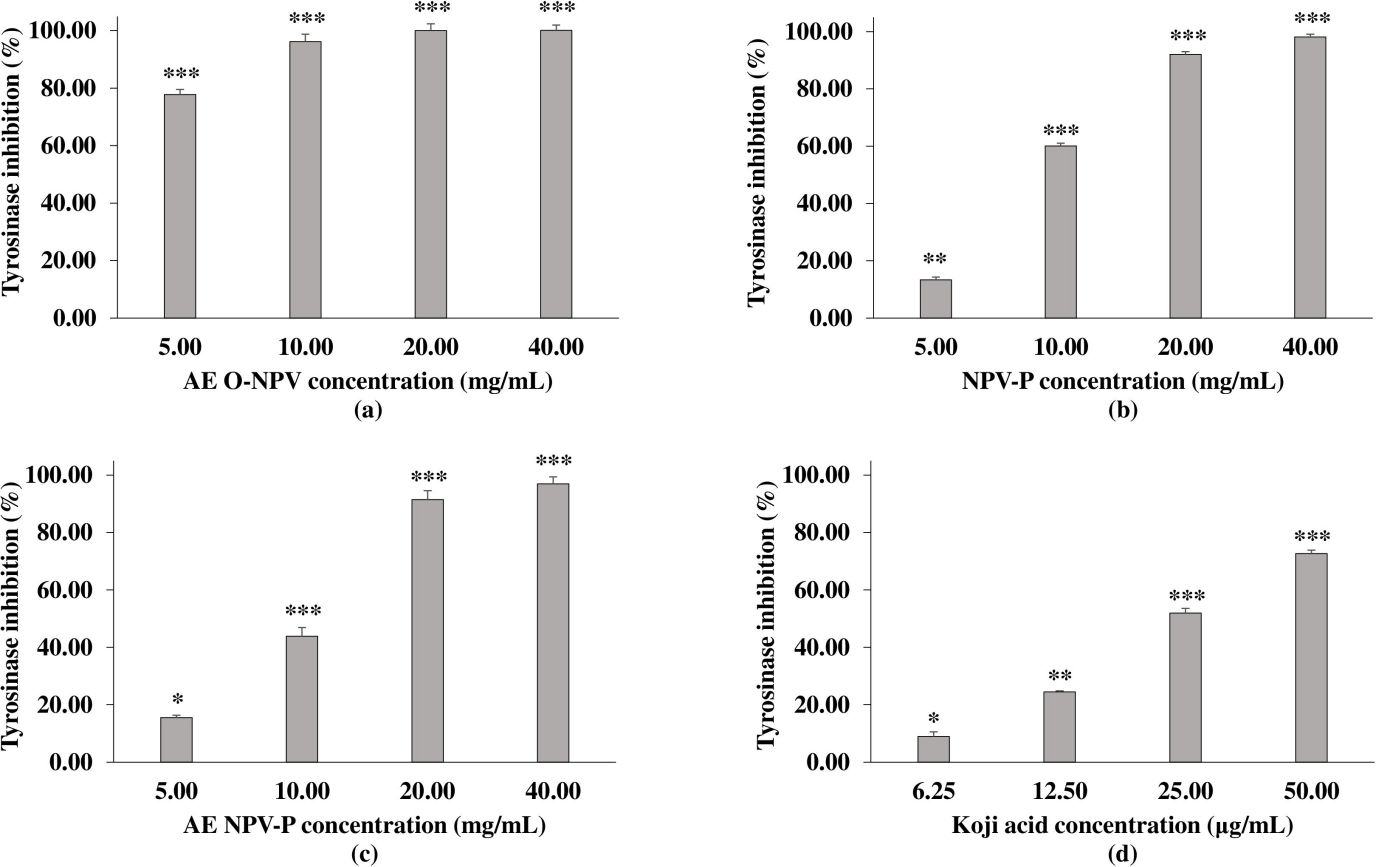
Anti-mushroom tyrosinase activity of (A) AE O-NPV, (B) NPV-P and (C) AE NPV-P at concentrations of 5.00, 10.00, 20.00 and 40.00 mg/mL. Kojic acid served as a tyrosinase inhibitor. Each value represents the mean ± standard deviation (*n* = 3).^∗^*p* < 0.05,^∗∗^*p* < 0.01, ^∗∗∗^*p* < 0.001.

Tyrosinase is a key enzyme that catalyzes the rate-limiting step in melanin synthesis (Hutchinson et al., 2019). For the anti-mushroom tyrosinase activity, the sample were tested using L-DOPA as a substrate. Figure 3 shows the significant concentration–response inhibitory effect of the AE O-NPV, NPV-P and AE NPV-P compared to the control (without treatment). The results demonstrated that all concentrations of the AE O-NPV showed a tyrosinase inhibitory activity higher than 50% at *p* <0.001. The order of the inhibitory potency, as evaluated by the half-inhibition concentration, or IC_50_, was 4.00 ± 0.04 mg/mL (AE O-NPV) >9.51 ± 0.04 mg/mL (NPV-P) >10.57 ± 0.12 mg/mL (AE NPV-P). Thus, the AE O-NPV possessed the most potent anti-mushroom tyrosinase activity which was related to the amount of phenolic and flavonoid contents as well as the ABTS radical scavenging activity. Kojic acid served as a tyrosinase inhibitor, showing an IC_50_ value of approximately 33.55 ± 1.69 µg/mL. Therefore, the AE O-NPV, NPV-P and AE NPV-P showed mushroom tyrosinase inhibition, which was selected to investigate the chemical compounds using high–performance liquid chromatography.

### High-performance liquid chromatography (HPLC) analysis

In this study, there were seven standards of phenolic compounds, including gallic acid, protocatechuic acid, vanillic acid, caffeic acid, coumaric acid, ferulic acid and sinapic acid, while catechin, rutin and quercetin served as the standards of the flavonoids (Figs. S1A, S2A). The amount of phenolic and flavonoid compounds is shown in Table 2. The HPLC data revealed that vanillic acid as a phenolic acid (42.13 ± 1.56 µg/g dry weight) was only found in the AE O-NPV (Fig. S1B). Among the three flavonoids identified in the AE O-NPV, NPV-P and AE NPV-P, rutin was found in all samples (Fig. S2B). The AE O-NPV contained the highest amount of rutin, followed by the AE NPV-P and NPV-P, whereas catechin was present in the AE O-NPV (12.92 ± 2.97 µg/g dry weight) and NPV-P (1.33 ± 0.24 µg/g dry weight). However, quercetin was only shown in the NPV-P, which was not obtained from samples using liquid–liquid extraction (Fig. S2B).

**Table 2:** Quantification of the identified individual phenolic and flavonoid compounds of nipa palm vinegar using an HPLC system.

**Identified compound**	**Quantity (µg/g dry weight)**		
	**AE O-NPV**	**NPV-P**	**AE NPV-P**
Vanillic acid	42.13 ± 1.56	ND	ND
Catechin	12.92 ± 2.97	1.33 ± 0.24	ND
Rutin	52.00 ± 7.79	1.84 ± 0.87	50.83 ± 7.43
Quercetin	ND	1.59 ± 0.10	ND

**Notes.**

Each value is presented as the mean ± standard error of the mean.

ND, not detected.

### Molecular docking analysis and molecular dynamics simulation of the studied compounds against mushroom tyrosinase

**Figure 4:**
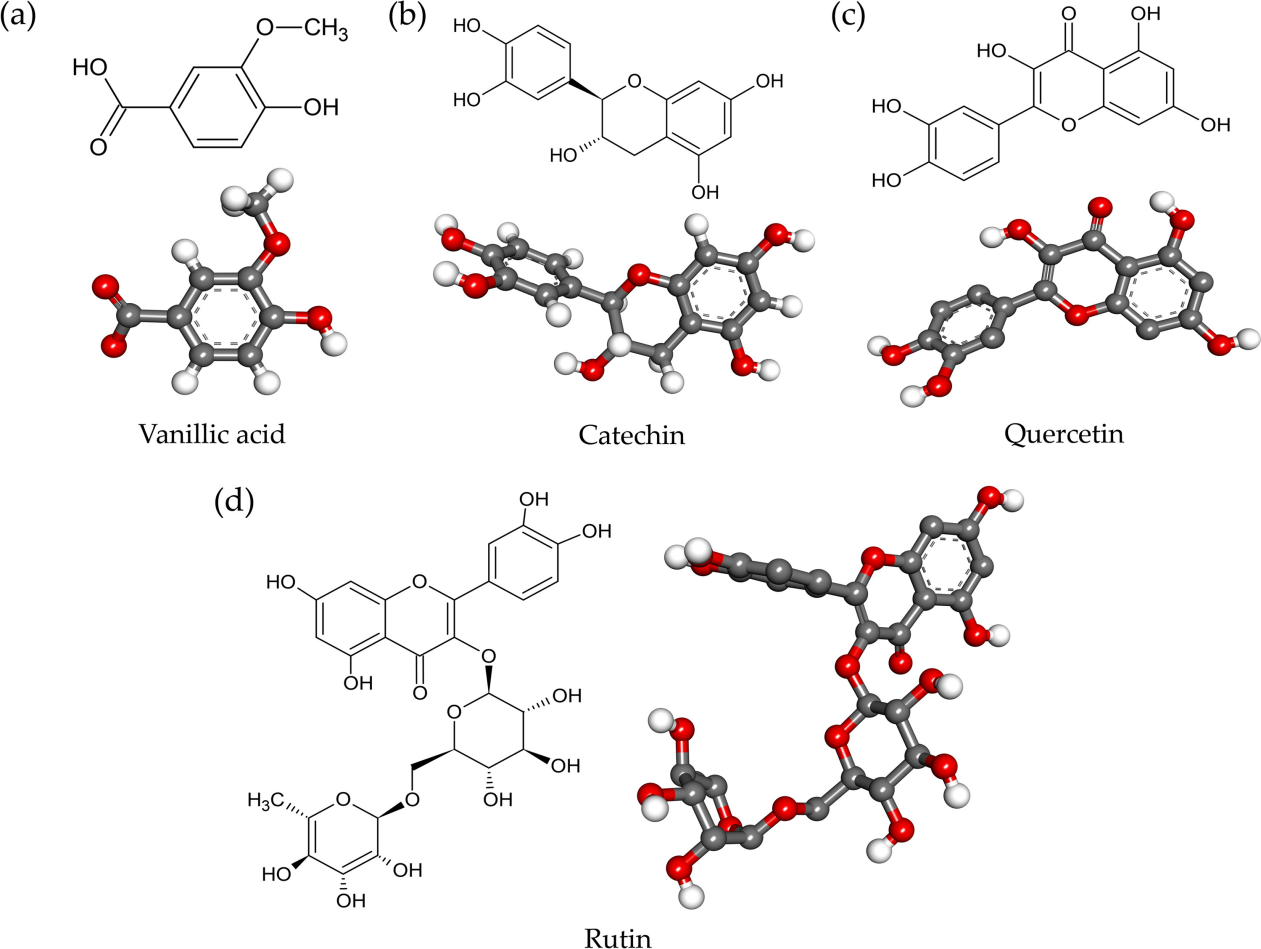
The 2D and 3D chemical structures of (A) vanillic acid; (B) catechin; (C) quercetin; and (D) rutin.

**Figure 5:**
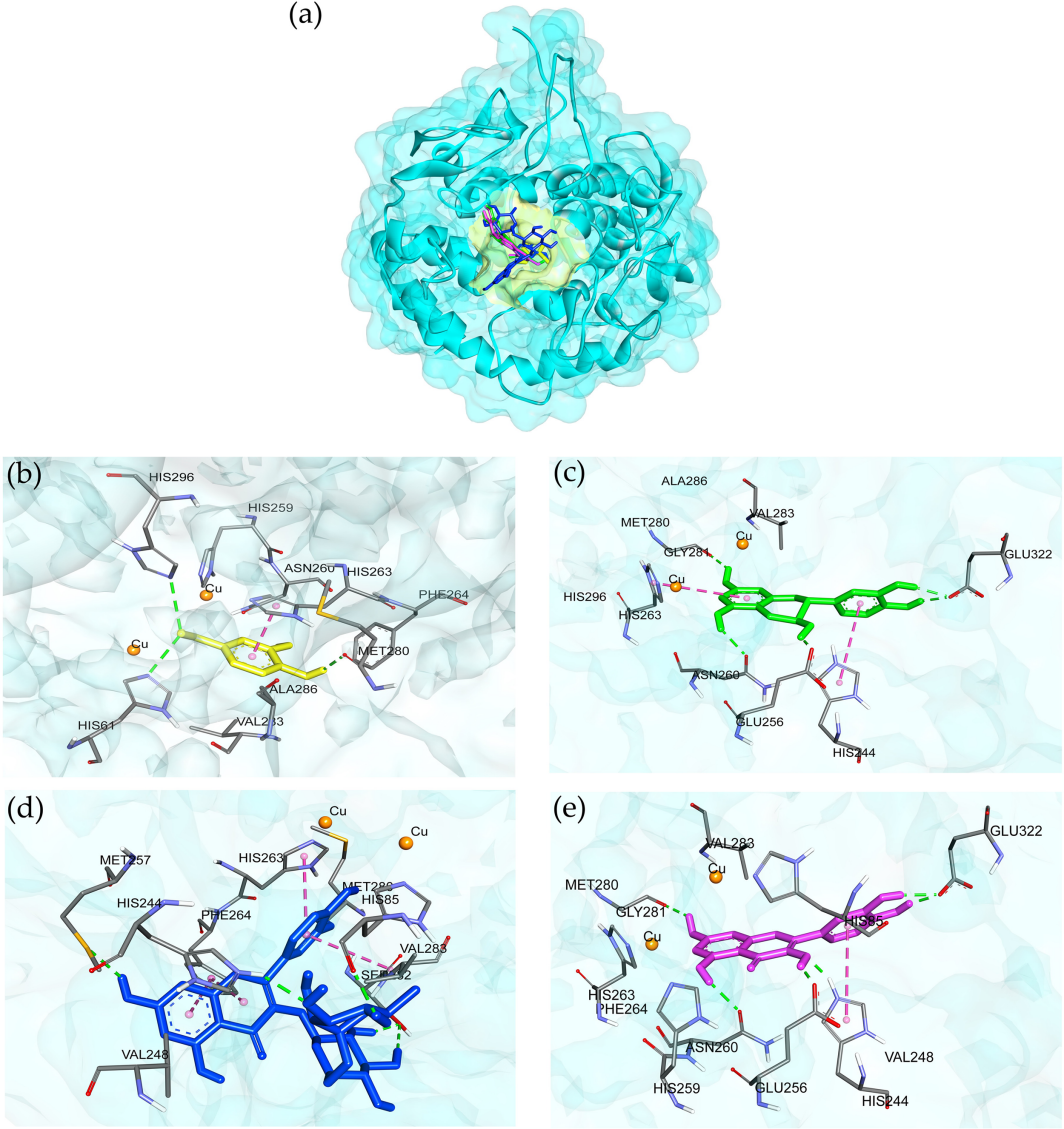
3D protein–ligand interactions at the tyrosinase active site of the selected compounds from the AE O-NPV, NPV-P and AE NPV-P. (A) The binding position of four compounds at active sites of tyrosinase; these compounds were (B) vanillic acid (yellow), (C) catechin (green), (D) rutin (blue), and (E) quercetin (pink) which interacted with the tyrosinase close to the copper atoms (orange). The green, dashed lines represent hydrogen bonding; the pink, dashed lines represented *π*–*π* interactions.

Four ligands from the AE O-NPV, NPV-AE and AE NPV-P as well as five control inhibitors were docked against the tyrosinase targets in this docking study. The 2D and optimized 3D structures of the ligands from the extracts are shown in Fig. 4. As shown in Table 3, the binding energies, hydrogen bonds with histidine residues and other amino acids resulted from the docking experiment. When docked with tyrosinase, vanillic acid from the AE O-NPV produced the utmost binding energy of −8.69 kcal/mol, which was better than the other compounds and all of the selected positive inhibitors. Moreover, the binding energy of catechin (binding energy at −6.02 kcal/mol) and quercetin (binding energy at −6.06 kcal/mol) was also better than that of the selected positive inhibitors. The binding position of the four compounds were located at the active sites of tyrosinase (Fig. 5A). The docked catechin, quercetin and rutin are shown in Figs. 4B–4D. Additional similar hydrogen-bonding and hydrophobic interactions with HIS residues and other amino acids were found in their docked conformations. The vanillic acid compound was found to fit into the active binding site and interacted with specific catalytic HIS residues by forming hydrogen bonds, including HIS61 and HIS296 (Table 3 and Fig. 5B). Vanillic acid showed a smaller molecular weight than the other compounds. Therefore, vanillic acid might fit and interfere with the interaction between the cofactor copper molecules in the catalytic HIS residues, leading to the inhibition of the tyrosinase function. Vanillic acid formed hydrophobic interactions by *π*–*π* stacking at the active site residues with HIS263 (Fig. 5B). All of these interactions facilitated the anchor of vanillic acid in the binding site of tyrosinase.

**Table 3:** Docking results of the compounds from the AE O-NPV, NPV-P and AE NPV-P against mushroom tyrosinase (PDB ID: 2Y9X).

**Compound Name**	**Formula**	**PubChem ID**	**Tyrosinase**			
			**Binding Energy** (**kcal**/**mol**)	**Inhibition constant**	**Active site** ** HIS residues involved** ** in H** **-** **Bond**	**Active site** ** Other residues involved** ** in H** **-** **Bond**
Vanillic acid	C_8_H_8_O_4_	8,468	−8.69	428.75 nM	HIS61, HIS296	MET280
Catechin	C_15_H_14_O_6_	9,064	−6.02	38.95 µM	ND	GLU256, ASN260, GLY281, GLU322
Rutin	C_27_H_30_O_16_	5,280,805	−5.95	43.57 µM	HIS85, HIS244	MET257, SER282
Quercetin	C_15_H_10_O_7_	5,280,343	−6.06	36.37 µM	HIS244	GLU256, ASN260, GLU322, GLY281
**Control Inhibitors**						
Aloin	C_21_H_22_O_9_	12,305,761	−5.77	59.12 µM	HIS85	ASN81, CYS83, GLU322, ASN320
Deoxyarbutin	C_11_H_14_O_3_	11,745,519	−5.32	125.01 µM	ND	ASN320, GLU322
Arbutin	C_12_H_16_O_7_	440,936	−4.62	410.88 µM	HIS85	CYS83, ALA323, ASN81
Kojic acid	C_6_H_6_O_4_	3,840	−4.42	573.31 µM	HIS85	GLU322
Hexylresorcinol	C_12_H_18_O_2_	3,610	−4.83	288.36 µM	ND	GLY281, VAL283

**Figure 6:**
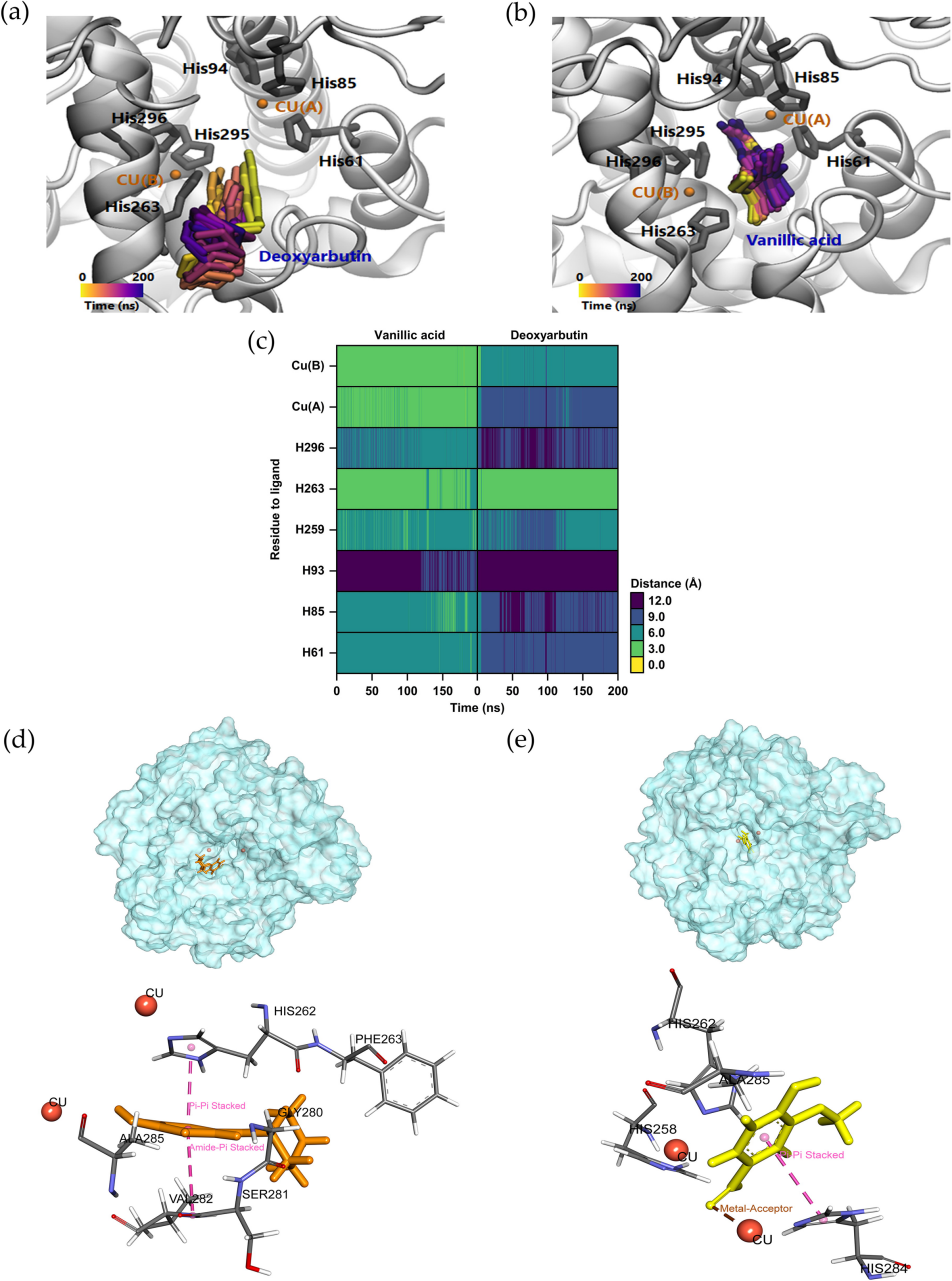
Molecular dynamic simulation results of the deoxyarbutin and vanillic acid to the binding pocket area of tyrosinase. Trajectory timestep of deoxyarbutin (A) and vanillic acid ligand (B) in the pocket site *versus* the MD simulation time (200 ns), (C) the distances of residue to ligand from trajectory timestep analysis, (D) Rg plots between ligands and tyrosinase binding residues along 200 ns simulation, (E and F) Binding area comparison between bioactive vanillic acid compounds and deoxyarbutin in tyrosinase binding site. Pink lines represent *π*–*π* stacking hydrophobic interactions and brown lines represent metal-acceptor interactions.

Earlier studies have indicated that vanillic acid exhibits a strong preference for the tyrosinase inhibitor with the highest binding energy and interacts with the catalytic residues (Girawale et al., 2022). To assess the stability of the conformations in comparison to deoxyarbutin (positive control), MD simulations were carried out in water solvent at physiological conditions. In our study, the MD simulations were conducted in an isotonic 0.15 M NaCl solution at a temperature of 25 ° C (298.15 K) and pH 7 to replicate physiological conditions. Over the course of the 200 ns simulation, the trajectory analysis of ligands within the tyrosinase pocket confirmed the stability of vanillic acid and deoxyarbutin within the enzyme’s active site. This stability was evident as they formed close and consistent interactions with crucial histidine and copper atoms, as visually demonstrated in Figs. 6A and 6B. Furthermore, we quantitatively assessed the distances between these key histidine residues and copper atoms in relation to the ligands. Notably, vanillic acid exhibited particularly close proximity to copper A and B, as well as HIS263, with distances measuring less than 3 angstroms, as illustrated in Fig. 6C. In this study, the SIE method was utilized to determine the binding energies of two tyrosinase inhibitors, namely vanillic acid and deoxyarbutin. The results showed that the binding energy of vanillic acid (at −6.43 ± 0.37 kcal/mol) was higher than that of deoxyarbutin (at −5.23 ± 0.29 kcal/mol). Furthermore, it was observed that vanillic acid exhibited robust interactions with the tyrosinase complex, primarily attributed to the van der Waals force with an energy of −22.48 ± 2.01 kcal/mol, representing the non-coulombic component as indicated in Table 4. Vanillic acid and deoxyarbutin bind to the active site of tyrosinase, with vanillic acid binding stably and close to copper molecules. Vanillic acid interacts with HIS284 through *π*–*π* stacking hydrophobic interactions and forms a metal-acceptor interaction with the copper molecule at the active site (Fig. 6D). In contrast, deoxyarbutin interacts with HIS262 and VAL282 through *π*–*π* stacking hydrophobic interactions but does not interact with the copper molecule (Fig. 6E). These observations suggest that vanillic acid has a preferred location in the binding pocket and is likely to be a more effective inhibitor of tyrosinase.

**Table 4:** Evaluation of binding affinity of tyrosinase and ligands from MD simulations using the solvent interaction energy (SIE) analysis.

**Ligands**	Δ*G*_**SIE**_ (kcal/mol)	${\Delta E}_{\mathbf{SIE}}^{\mathbf{V DW}}$ (kcal/mol)	${\Delta E}_{\mathbf{SIE}}^{\mathbf{coulombic}}$ ** (kcal/mol)**
Deoxyarbutin	−5.23 ± 0.29	−24.17 ± 2.02	−2.55 ± 0.96
Vanillic acid	−6.43 ± 0.37	−22.48 ± 2.01	16.00 ± 3.95

**Notes.**

Δ G_**SIE,**_binding energy E^vdw^ van der Waals contribution E^coulombic^ coulombic contribution

### Structure-activity relationship of tyrosinase inhibitory activity

**Figure 7:**
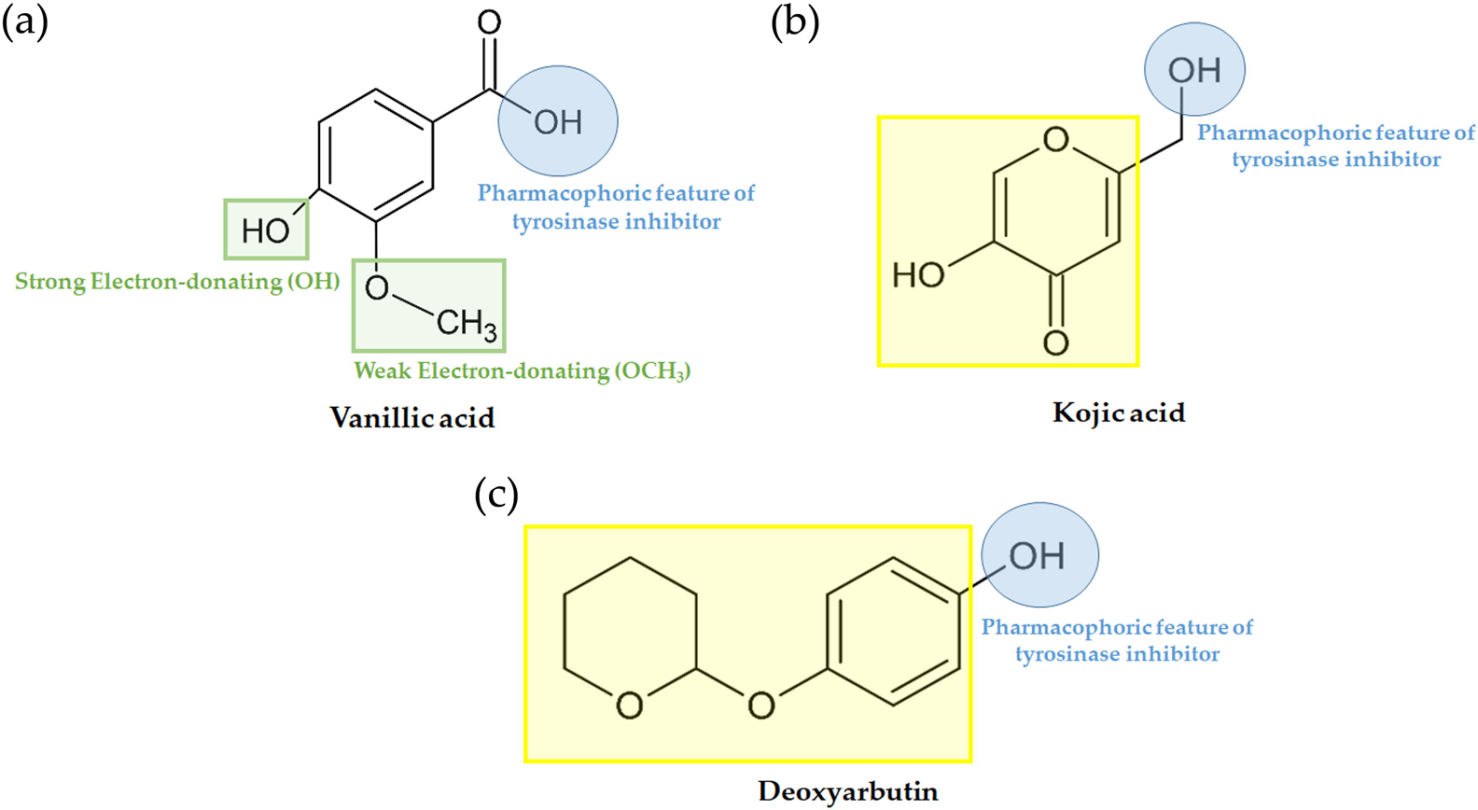
Pharmacophoric features of (A) vanillic acid, (B) kojic acid and (C) deoxyarbutin.

In our current investigation, we delved into the anti-tyrosinase properties of the most potent ligands. This analysis extended to examining their shared structural-activity relationship with various chemical descriptors. Furthermore, we harnessed Deep Convolutional Neural Networks (DCNNs) through KDEEP (https://www.playmolecule.com/Kdeep/) to predict both the pIC_50_ and binding affinity (pKd) of the protein-ligand complex. We also conducted an assessment of structural features, encompassing hydrophobicity, aromaticity, hydrogen-bond donating and accepting capabilities, as well as positive and negative ionizability, metallic character, and total excluded volume. These features were calculated and compared to pre-existing data derived from the PDBbind v.2016 database. The alignment of experimental tyrosinase inhibition assay results with computational findings is presented in Table 5. Vanillic acid exhibited a pIC50 and binding affinity (pKd) of approximately 4.36 µM, which is in close proximity to that of standard inhibitors such as deoxyarbutin and kojic acid. However, prior experimental tyrosinase inhibition assays for vanillic acid reported a significantly higher value of 15.84 mM (Girawale et al., 2022). Furthermore, there has been limited exploration of the structure–activity relationship (SAR) focusing on the distinct positions of the hydroxyl group within the pharmacophoric feature of tyrosinase inhibitors, as compared to kojic acid and deoxyarbutin (Fig. 7).

### Chemoinformatics, Lipinski’s rule of five (RO5), skin absorption and toxicity assessment

The SwissADME and pkCSM tools were used to perform the predict pharmacokinetics properties, such as the number of hydrogen bond acceptors/donors, solubility, polar surface area and bioavailability score. The computational results predicted that the molecular weight of vanillic acid, catechin and quercetin were less than 500 g/mol, which correlated to the reported literature regarding the standard range for the molecular weight (160–500) (Tian et al., 2015). Furthermore, vanillic acid, catechin and quercetin passed Lipinski’s rule of five (RO5) by possessing an acceptable number of HBA, HBD, LogP, and PSA (Table S1). Moreover, vanillic acid showed good skin permeability (Table 6). All of the selected compounds from the AE O-NPV, NPV-P and AE NPV-P might not cause AMES toxicity, hepatotoxicity and skin sensitization (Table 6).

**Table 5:** The relationship of experimental tyrosinase inhibition and computational prediction of vanillic acid and tyrosinase inhibitors.

**Compound name**	**Experimental tyrosinase inhibition assays**	**Computational prediction**			
	**IC**_**50**_ **values** ** (** **µM** **)**	**dG** ** (** **kcal** **/** **mol** **)**	**Lig** **.** ** Efficiency** ** (** **kcal** **/** **mol** **)**	**pIC**_**50**_ **(** **µM** **)**	**pKd** ** (** **µM** **)**
Vanillic acid	15.84 mM (Girawale et al., 2022)	−5.88	−0.49	4.36	4.36
Deoxyarbutin	57.70 µM (Chawla et al., 2008)	−4.44	−0.32	3.37	3.29
Kojic acid	40.69 µM (Chen et al., 2015)	−4.91	−0.49	3.59	3.64

**Table 6:** Skin absorption and toxicity assessment of natural products from AE O-NPV, NPV-P and AE NPV-P predicted by SwissADME and pkCSM server.

**Compound name**	**Skin Permeability (log Kp**≥−7.0 cm**/s)**	**Toxicity**		
		**AMES toxicity**	**Hepatotoxicity**	**Skin sensitization**
Vanillic acid	−6.31	No	No	No
Catechin	−7.82	No	No	No
Quercetin	−7.05	No	No	No
Rutin	−10.26	No	No	No
**Control inhibitors**				
Aloin	−8.94	No	No	No
Deoxyarbutin	−5.78	No	No	No
Arbutin	−8.92	No	No	No
Kojic acid	−7.62	No	No	No
Hexylresorcinol	−5.04	No	No	Yes

## Discussion

Phenolic and flavonoids are important constituents in plants and can be tyrosinase inhibitors, such as copper chelators (Panzella & Napolitano, 2019). Liquid–liquid extraction is a common method used to separate phenolic compounds (Silva, García & Ottens, 2018). The highest phenolic content was obtained by liquid–liquid extraction in the original nipa palm vinegar. Our result showed that the least phenolic content was found in the nipa palm vinegar powder that was obtained from the aqueous layer using liquid–liquid extraction. There was a significant difference between the AE O-NPV, NPV-P and AE NPV-P. The same as our result, Palachum, Klangbud & Chisti (2022) reported that the total phenolic compound of spray-dried nipa palm vinegar powder was 1.05 ± 0.02 mg GAE/g. Compared to a previous study, Sudjaroen et al. (2021) reported a total phenolic content of 1.76 ± 0.02 mg GAE/g in an aqueous extract of nipa palm vinegar. Senghoi & Klangbud (2021) evaluated the total phenolic content in nipa palm vinegar from three cultivated areas, which ranged between 80.31 ± 4.00 and 177.16 ± 0.95 µg GAE/mL. Saithong, Nitipan & Permpool (2019) found that the phenolic contents of nipa sap from raw material, local market and surface culture fermentation were 448.73 ± 0.58, 316.23 ± 0.76 and 253.98 ± 0.14 µg GAE/mL. Chatatikun & Kwanhian (2020) determined a phenolic content of 167.10 ± 10.15 µg GAE/mL of nipa palm vinegar. In our study, AE O-NPV, NPV-P and AE NPV-P had high amounts of phenolic contents, which were affected by the different extracts, areas and methods (Alara, Abdurahman & Ukaegbu, 2021; Senghoi & Klangbud, 2021).

Flavonoids are major polyphenolic groups that are found in fruits, vegetables, cereals and beverages. Flavonoids have potent antioxidant properties that can stabilize free radicals or reactive oxygen species (El-Nashar et al., 2021). Senghoi & Klangbud (2021) described nipa palm vinegar from three cultivated areas with different sources (varied range from 1.60 to 2.27 µg QE/mL). The nipa palm syrup from three different plantation areas presented flavonoid contents ranging from 16.3 to 27.2 mg rutin equivalents (RE)/100 g dw (Saengkrajang, Chaijan & Panpipat, 2021). As mentioned above, the amount of flavonoid compounds depend on the geographic origin or plantation area. The extraction method of liquid–liquid extraction is a common technique for separating flavonoid from plants and also affects the flavonoid content (Chávez-González et al., 2020). The present results show high flavonoid contents in the AE O-NPV, NPV-P and AE NPV-P. However, the flavonoid content of the spray-dried samples, such as the NPV-P and AE NPV-P, were lower than the AE O-NPV, which was obtained from fresh nipa palm vinegar.

The present study determined the antioxidant activity of the AE O-NPV, NPV-P and AE NPV-P using DPPH and ABTS assays. Both methods are based on electron transfers and the reduction of color oxidants. The DPPH assay is based on the reduction of purple DPPH ^•^ to 1,1-diphenyl-2-picryl hydrazine, whereas the ABTS assay depends on the generation of a blue/green ABTS ^•+^. The ABTS^•+^ radical cation can be reduced by antioxidants in plants, fruits, vegetables and beverage (Floegel et al., 2011). In a previous study, the DPPH scavenging activity values of nipa palm vinegar at concentration of 25 µL/mL in three different areas was not more than 20% scavenging activity as well as ABTS ^•+^ scavenging activity (lower than 14% scavenging activity) (Senghoi & Klangbud, 2021). However, the ethyl acetate and aqueous extracts of nipa palm vinegar exhibited high antioxidant activity using the DPPH assay, but they showed low ABTS scavenging activity (Yusoff et al., 2015b). Our present study shows that NPV-P and AE NPV-P are products from the powder of nipa palm vinegar, which had low DPPH^•^ scavenging activity, while only the AE O-NPV had the scavenging ability of the ABTS radical cation.

An HPLC analysis is commonly used to separate and identify the phenolic acids and flavonoids from a plant sample (Kuppusamy et al., 2018). Previously, we reported that nipa palm vinegar contained gallic acid, catechin, rutin, isoquercetin and quercetin (Chatatikun & Kwanhian, 2020), while the chemical profiling analysis of the aqueous extract of nipa palm vinegar using gas chromatography-mass spectrometry (GC-MS) revealed five compounds containing acetic acid; 2,3-butanediol; 1-(2-butoxyethyoxy)-ethanol; 5-bromo-2-hydroxybenzaldehyde; and (4-aminophenyl)-phenylmethanone (Yusoff et al., 2015b). Moreover, the nutritional analysis of the nipa palm vinegar powder showed vitamins A, B1, B2 and C as well as acetic acid and phenolic contents (Palachum, Klangbud & Chisti, 2022). In the present study, it was noted that nipa palm vinegar contained vanillic acid, catechin, rutin and quercetin. We chose these compounds to perform a molecular docking study against mushroom tyrosinase, skin absorption and toxicity assessment.

The enzyme tyrosinase is a metalloenzyme, which performs specific functions in melanogenesis (Videira, Moura & Magina, 2013). At the active site of tyrosinase, a binuclear copper site interacts with histidine residues and the conserved residues in the core region. The first copper ion (CuA) interacts with the HIS61, HIS85 and HIS94 residues, and the second copper ion (CuB) is coordinated with HIS259, HIS263 and HIS296 (Iraji et al., 2020). Previous studies revealed that ASN81, ASN260, HIS259, HIS263 and MET280 are the crucial residues responsible for stabilizing the protein inhibitor complex (Pintus et al., 2017), therefore trapping the histidine amino acid coordinated with copper ion, which plays an important role in tyrosinase inhibition. Tyrosinase inhibitors, such as arbutin and kojic acid, have been tested in pharmaceuticals and cosmetics for their capability of preventing the overproduction of melanin (Kim & Uyama, 2005). Previously, vanillic acid showed a docked score of −5.8 kcal/mol against tyrosinase, but it did not form hydrogen bonding to the active site residues (Mechqoq et al., 2022). Ligand optimization, the addition of missing hydrogen atoms and the assignment of charges were necessary processes for the molecular docking. Based on a series of beneficial interactions with the enzyme and its cofactors (such as copper ion), active substances were generally predicted to exhibit high affinity for the active site. The isookanin compound binding mode lacked interactions with copper ions in the tyrosinase, which resulted in a lower docking score than the chelators of copper ions, such as luteolin, kaempferol, quercetin and robinetin compounds (Jakimiuk et al., 2022). Vanillic acid produced the strongest binding score and formed hydrogen bonding at the active site residues containing copper molecules.

Vanillic acid compound was found to fit into the active binding site and interacted with specific catalytic HIS residues by forming hydrogen bonds, including HIS61 and HIS296 (Table 3 and Fig. 5B). Vanillic acid showed a smaller molecular weight than the other compounds. Therefore, vanillic acid might fit and interfere with the interaction between the cofactor copper molecules in the catalytic HIS residues, leading to the inhibition of the tyrosinase function. Vanillic acid formed hydrophobic interactions by *π*–*π* stacking at the active site residues with HIS263 (Fig. 5B). All of these interactions facilitated the anchor of vanillic acid in the binding site of tyrosinase. Novel inhibitors of the mushroom tyrosinase enzyme were previously developed, synthesized, and validated through *in vitro* and *in silico* studies. These inhibitors include 2-phenylchromone derivatives (Ashraf et al., 2021), 3-hydroxyflavone (Ashraf et al., 2020), as well as thioflavones and thioflavonols (Mughal et al., 2022). They effectively inhibited tyrosinase activity by binding to essential amino acids and the copper atoms in the active site of the enzyme. To verify the docking result, molecular dynamic simulation was utilized. The analysis of ligands within the binding pocket of tyrosinase revealed the stability of the vanillic acid and deoxyarbutin ligand-protein complex. This stability was observed through the trajectory timestep analysis, as depicted in Figs. 6A–Figs. 6C. In Fig. 6C, vanillic acid exhibited greater proximity to copper A and B, as well as HIS263, with distances measuring less than 3 angstroms when compared to deoxyarbutin. This dinuclear copper-containing metalloenzyme is abundant in nature and plays a pivotal role in melanin pigment biosynthesis (Obaid et al., 2021). Researchers aimed to design compounds by adding tyrosinase inhibitor functional groups that irreversibly bind to binuclear copper ions. Thioflavones and thioflavonols were synthesized with added sulfur atoms, forming connections with receptor backbones consisting of histidine, phenylalanine, alanine, and active-site copper ions in tyrosinase. This process led to the ultimate inhibition of tyrosinase activity (Mughal et al., 2022). The main forces involved in the interaction between vanillic acid and tyrosinase were van der Waals forces, *π*- *π* stacking, and a metal-acceptor interaction with the copper molecule at the active site of the tyrosinase protein. These interactions were able to maintain the stability of the vanillic acid-tyrosinase complex during the simulation process. A prior study showed that calycosin primarily binds to tyrosinase through the contribution of van der Waals forces, as evidenced by the formation of hydrophobic interactions observed during the molecular dynamic simulation at 100 ns (Tayier et al., 2021).

On this basis, the molecular docking and molecular dynamic simulation results provide a rational explanation for the interactions between the compounds from the nipa palm vinegar (*i.e.,* AE O-NPV, NPV-P and AE NPV-P) and tyrosinase, providing valuable information for the development of tyrosinase inhibitors and for cosmetic use. Computational predictions of vanillic acid’s pIC_50_ and pKd indicated similarities to deoxyarbutin and kojic acid (Table 5). However, in previous studies, vanillic acid exhibited inhibitory activity against *in vitro* tyrosinase with an IC_50_ of 15.84 mM (Girawale et al., 2022), which was lower than that of the reference standard inhibitors, deoxyarbutin and kojic acid (Table 5). This difference could be attributed to the use of human tyrosinase in the *in vitro* assay, while our predictions were based on mushroom tyrosinase. Consequently, it is advisable to conduct *in vitro* antityrosinase activity testing of vanillic acid using mushroom tyrosinase. Vanillic acid exhibited distinct pharmacophoric attributes, featuring two potent electron-donating hydroxy groups and one less potent electron-donating methoxy group (Fig. 7A). Notably, vanillic acid shared these pharmacophoric characteristics with known tyrosinase inhibitors, kojic acid (Fig. 7B) and deoxyarbutin (Fig. 7C), both of which also presented two hydroxy groups at equivalent positions. These shared features are likely to have a significant impact on the observed activities, as supported by experimental and computational investigations (Ashraf et al., 2020; Obaid et al., 2021). To enhance the anti-tyrosinase efficacy of vanillic acid, we propose structural modifications involving the incorporation of functional groups like F, Cl, Br, CH_3_, OH, and OMe. These functional groups were demonstrated to play a pivotal role in the activity of potent tyrosinase inhibitors, specifically in 2-phenylchromone derivatives, as reported in a prior study (Ashraf et al., 2021). Moreover, vanillic acid, catechin and quercetin passed Lipinski’s rule of five. Our study showed that all of the selected compound from the nipa palm vinegar might not cause AMES toxicity, hepatoxicity and skin sensitization. Vanillic acid showed the best skin permeability. From our results, nipa palm vinegar could be a new ingredient for tyrosinase inhibition.

## Conclusions

Nipa palm vinegar and its extracts posse concentration-dependent anti-tyrosinase activity as well as antioxidant activity. The HPLC analysis of nipa palm vinegar and its extracts proves the presence of phenolics and flavonoids as active compounds. The molecular docking studies and molecular dynamic simulation agreed with the observed in the result of anti-mushroom activity, in which the anti-tyrosinase activity of vanillic acid was more stable binding to tyrosinase than positive control through the tyrosinase hydrophobic binding pocket surrounding the copper active site. Hence, nipa palm vinegar represented a source of phytochemical compounds with potential use for treating hyperpigmentation in pharmaceutical and cosmetic products. Further research is needed to elucidate the anti-melanogenic activity of nipa palm vinegar and its active compounds through molecular mechanism.

##  Supplemental Information

10.7717/peerj.16494/supp-1 Supplemental Information 1 Chemo-informatics assessment of natural products from AE O-NPV, NPV-P and AE NPV-P predicted by SwissADME server

10.7717/peerj.16494/supp-2 Supplemental Information 2 Structure–activity relationship (SAR) of the most potent compounds

10.7717/peerj.16494/supp-3 Supplemental Information 3 HPLC chromatogram of (A) six reference standards of phenolic acids and (B) AE O-NPV at 280 nm

10.7717/peerj.16494/supp-4 Supplemental Information 4 HPLC chromatogram of (A) three flavonoid reference standards, (B) AE O-NPV, (C) NPV-P, and (D) AE NPV-P at 280 nm

10.7717/peerj.16494/supp-5 Supplemental Information 5 Raw data

## Additional Information and Declarations

### Data Availability

The following information was supplied regarding data availability: The raw measurements are available in the Supplemental Files.

## References

[ref-1] Alara OR, Abdurahman NH, Ukaegbu CI (2021). Extraction of phenolic compounds: a review. Current Research in Food Science.

[ref-2] Alshaye NA, Mughal EU, Elkaeed EB, Ashraf Z, Kehili S, Nazir Y, Naeem N, Abdul Majeed N, Sadiq A (2023). Synthesis and biological evaluation of substituted aurone derivatives as potential tyrosinase inhibitors: in vitro, kinetic, QSAR, docking and drug-likeness studies. Journal of Biomolecular Structure & Dynamics.

[ref-3] Aremu OO, Oyedeji AO, Oyedeji OO, Nkeh-Chungag BN, Rusike CRS (2019). In vitro and *In vivo* antioxidant properties of *Taraxacum officinale* in N *ω*-nitro—L-arginine methyl ester (L-NAME)-induced hypertensive rats. Antioxidants.

[ref-4] Ashraf J, Mughal EU, Alsantali RI, Obaid RJ, Sadiq A, Naeem N, Ali A, Massadaq A, Javed Q, Javid A, Sumrra SH, Zafar MN, Ahmed SA (2021). Structure-based designing and synthesis of 2-phenylchromone derivatives as potent tyrosinase inhibitors: *In vitro* and *in silico* studies. Bioorganic & Medicinal Chemistry.

[ref-5] Ashraf J, Mughal EU, Sadiq A, Bibi M, Naeem N, Ali A, Massadaq A, Fatima N, Javid A, Zafar MN, Khan BA, Nazar MF, Mumtaz A, Tahir MN, Mirzaei M (2020). Exploring 3-hydroxyflavone scaffolds as mushroom tyrosinase inhibitors: synthesis, X-ray crystallography, antimicrobial, fluorescence behaviour, structure–activity relationship and molecular modelling studies. Journal of Biomolecular Structure & Dynamics.

[ref-6] Burnett CL, Bergfeld WF, Belsito DV, Hill RA, Klaassen CD, Liebler DC, Marks JG, Shank RC, Slaga TJ, Snyder PW, Andersen FA (2010). Final report of the safety assessment of Kojic acid as used in cosmetics. International Journal of Toxicology.

[ref-7] Chatatikun M, Kwanhian W (2020). Phenolic profile of nipa palm vinegar and evaluation of its antilipidemic activities. Evidence-Based Complementary and Alternative Medicine.

[ref-8] Chávez-González ML, Sepúlveda L, Verma DK, Luna-García HA, Rodríguez-Durán LV, Ilina A, Aguilar CN (2020). Conventional and emerging extraction processes of flavonoids. Processes.

[ref-9] Chawla S, de Long MA, Visscher MO, Wickett RR, Manga P, Boissy RE (2008). Mechanism of tyrosinase inhibition by deoxyArbutin and its second-generation derivatives. British Journal of Dermatology.

[ref-10] Chen WC, Tseng TS, Hsiao NW, Lin YL, Wen ZH, Tsai CC, Lee YC, Lin HH, Tsai KC (2015). Discovery of highly potent tyrosinase inhibitor, T1, with significant anti-melanogenesis ability by zebrafish in vivo assay and computational molecular modeling. Scientific Reports.

[ref-11] Di Petrillo A, González-Paramás AM, Era B, Medda R, Pintus F, Santos-Buelga C, Fais A (2016). Tyrosinase inhibition and antioxidant properties of *Asphodelus microcarpus* extracts. BMC Complementary Alternative Medicine.

[ref-12] El Maaiden E, Qarah N, Ezzariai A, Mazar A, Nasser B, Moustaid K, Boukcim H, Hirich A, Kouisni L, El Kharrassi Y (2023). Ultrasound-Assisted Extraction of Isoquercetin from Ephedra alata (Decne): Optimization Using Response Surface Methodology and In Vitro Bioactivities. Antioxidants.

[ref-13] El-Nashar HAS, El-Din MIG, Hritcu L, Eldahshan OA (2021). Insights on the inhibitory power of flavonoids on tyrosinase activity: a survey from 2016 to 2021. Molecules.

[ref-14] Ferro S, Deri B, Germanò MP, Gitto R, Ielo L, Buemi MR, Certo G, Vittorio S, Rapisarda A, Pazy Y, Fishman A, De Luca L (2018). Targeting tyrosinase: development and structural insights of novel inhibitors bearing arylpiperidine and arylpiperazine fragments. Journal of Medicinal Chemistry.

[ref-15] Floegel A, Kim D-O, Chung S-J, Koo SI, Chun OK (2011). Comparison of ABTS/DPPH assays to measure antioxidant capacity in popular antioxidant-rich US foods. Journal of Food Composition and Analysis.

[ref-16] Gillbro J, Olsson M (2011). The melanogenesis and mechanisms of skin-lightening agents-existing and new approaches. International Journal of Cosmetic Science.

[ref-17] Girawale SD, Meena SN, Nandre VS, Waghmode SB, Kodam KM (2022). Biosynthesis of vanillic acid by Ochrobactrum anthropi and its applications. Bioorganic & Medicinal Chemistry.

[ref-18] Gullón B, Lú-Chau TA, Moreira MT, Lema JM, Eibes G (2017). Rutin: A review on extraction, identification and purification methods, biological activities and approaches to enhance its bioavailability. Trends in Food Science & Technology.

[ref-19] Hassan M, Shahzadi S, Kloczkowski A (2023). Tyrosinase inhibitors naturally present in plants and synthetic modifications of these natural products as anti-melanogenic agents: a review. Molecules.

[ref-20] Hossain M (2015). Utilization of mangrove forest plant: nipa palm (*Nypa fruticans* Wurmb). American Journal of Agriculture.

[ref-21] Hutchinson JA, Hamley IW, Edwards-Gayle CJC, Castelletto V, Piras C, Cramer R, Kowalczyk R, Seitsonen J, Ruokolainen J, Rambo RP (2019). Melanin production by tyrosinase activity on a tyrosine-rich peptide fragment and pH-dependent self-assembly of its lipidated analogue. Organic and Biomolecular Chemistry.

[ref-22] Iraji A, Adelpour T, Edraki N, Khoshneviszadeh M, Miri R, Khoshneviszadeh M (2020). Synthesis, biological evaluation and molecular docking analysis of vaniline-benzylidenehydrazine hybrids as potent tyrosinase inhibitors. BMC Chemistry.

[ref-23] Jakimiuk K, Sari S, Milewski R, Supuran CT, Şöhretoğlu D, Tomczyk M (2022). Flavonoids as tyrosinase inhibitors in in silico and in vitro models: basic framework of SAR using a statistical modelling approach. Journal of Enzyme Inhibition and Medicinal Chemistry.

[ref-24] Juliano CCA (2022). Spreading of dangerous skin-lightening products as a result of colourism: a review. Applied Sciences.

[ref-25] Kahkeshani N, Farzaei F, Fotouhi M, Alavi SS, Bahramsoltani R, Naseri R, Momtaz S, Abbasabadi Z, Rahimi R, Farzaei MH, Bishayee A (2019). Pharmacological effects of gallic acid in health and diseases: A mechanistic review. Iranian Journal of Basic Medical Sciences.

[ref-26] Kim YJ, Uyama H (2005). Tyrosinase inhibitors from natural and synthetic sources: structure, inhibition mechanism and perspective for the future. Cellular and Molecular Life Sciences.

[ref-27] Klangbud WK, Songsri J, Bunluepuech K, Chopjit P (2022). The efficacy of the traditional Thai remedy Ya-Ha-Rak against dengue virus type 2. Journal of Herbal Medicine.

[ref-28] Kooltheat N, Chujit K, Nuangnong K, Nokkaew N, Bunluepuech K, Yamasaki K, Chatatikun M (2021). Artemisia lactiflora extracts prevent inflammatory responses of human macrophages stimulated with charcoal pyrolysis smoke. Journal of Evidence -Based Integrative Medicine.

[ref-29] Kuppusamy P, Lee KD, Song CE, Ilavenil S, Srigopalram S, Arasu MV, Choi KC (2018). Quantification of major phenolic and flavonoid markers in forage crop Lolium multiflorum using HPLC-DAD. Revista Brasileira de Farmacognosia.

[ref-30] Laklaeng S-N, Kwanhian W (2020). Immunomodulation effect of *Nypa fruticans* palm vinegar. Walailak Journal of Science and Technology.

[ref-31] Lee AY (2021). Skin pigmentation abnormalities and their possible relationship with skin aging. International Journal of Molecular Sciences.

[ref-32] Lee SY, Baek N, Nam T-G (2016). Natural, semisynthetic and synthetic tyrosinase inhibitors. Journal of Enzyme Inhibition and Medicinal Chemistry.

[ref-33] Li P, Merz K (2016). MCPB.py: a python based metal center parameter builder. Journal of chemical information and modeling.

[ref-34] Liyanage A, Liyanage G, Sirimanna G, Schürer N (2022). Comparative study on depigmenting agents in skin of color. Journal of Clinical and Aesthetic Dermatology.

[ref-35] Mechqoq H, Hourfane S, El Yaagoubi M, Hamdaoui AEl, da Silva Almeida JRG, Rocha JM, El Aouad N (2022). Molecular docking, tyrosinase, collagenase, and elastase inhibition activities of Argan by-products. Cosmetics.

[ref-36] Mikami M, Sonoki T, Ito M, Funasaka Y, Suzuki T, Katagata Y (2013). Glycosylation of tyrosinase is a determinant of melanin production in cultured melanoma cells. Molecular Medicine Reports.

[ref-37] Mughal EU, Ashraf J, Hussein EM, Nazir Y, Alwuthaynani AS, Naeem N, Sadiq A, Alsantali RI, Ahmed SA (2022). Design, synthesis, and structural characterization of thioflavones and thioflavonols as potential tyrosinase inhibitors: *in vitro* and *in silico* studies. ACS Omega.

[ref-38] Obaid RJ, Mughal EU, Naeem N, Sadiq A, Alsantali RI, Jassas RS, Moussa Z, Ahmed SA (2021). Natural and synthetic flavonoid derivatives as new potential tyrosinase inhibitors: a systematic review. RSC Advances.

[ref-39] Palachum W, Klangbud WK, Chisti Y (2022). Spray-dried nipa palm vinegar powder: production and evaluation of physicochemical, nutritional, sensory, and storage aspects. Fermentation.

[ref-40] Panzella L, Napolitano A (2019). Natural and bioinspired phenolic compounds as tyrosinase inhibitors for the treatment of skin hyperpigmentation: recent advances. Cosmetics.

[ref-41] Peng Z, Wang G, He Y, Wang JJ, Zhao Y (2022). Tyrosinase inhibitory mechanism and anti-browning properties of novel kojic acid derivatives bearing aromatic aldehyde moiety. Current Research in Food Science.

[ref-42] Pintus F, Matos MJ, Vilar S, Hripcsak G, Varela C, Uriarte E, Santana L, Borges F, Medda R, Di Petrillo A, Era B, Fais A (2017). New insights into highly potent tyrosinase inhibitors based on 3-heteroarylcoumarins: anti-melanogenesis and antioxidant activities, and computational molecular modeling studies. Bioorganic & Medicinal Chemistry.

[ref-43] Pintus F, Spanò D, Corona A, Medda R (2015). Antityrosinase activity of Euphorbia characias extracts. PeerJ.

[ref-44] Plensdorf S, Livieratos M, Dada N (2017). Pigmentation disorders: diagnosis and management. American Family Physician.

[ref-45] Rai R, Karri R, Dubey K, Roy G (2022). Regulation of tyrosinase enzyme activity by glutathione peroxidase mimics. Journal of Agricultural and Food Chemistry.

[ref-46] Saengkrajang W, Chaijan M, Panpipat W (2021). Physicochemical properties and nutritional compositions of nipa palm (Nypa fruticans Wurmb) syrup. NFS Journal.

[ref-47] Saithong P, Nitipan S, Permpool J (2019). Optimization of vinegar production from nipa (*Nypa fruticans* Wurmb.) sap using surface culture fermentation process. Applied Food Biotechnology.

[ref-48] Senghoi W, Klangbud WK (2021). Antioxidants, inhibits the growth of foodborne pathogens and reduces nitric oxide activity in LPS-stimulated RAW 264.7 cells of nipa palm vinegar. PeerJ.

[ref-49] Shabir I, Kumar Pandey V, Shams R, Dar AH, Dash KK, Khan SA, Bashir I, Jeevarathinam G, Rusu AV, Esatbeyoglu T, Pandiselvam R (2022). Promising bioactive properties of quercetin for potential food applications and health benefits: A review. Frontiers in Nutrition.

[ref-50] Silva M, García JC, Ottens M (2018). Polyphenol liquid–liquid extraction process development using NRTL-SAC. Industrial & Engineering Chemistry Research.

[ref-51] Song Y, Chen S, Li L, Zeng Y, Hu X (2022). The hypopigmentation mechanism of tyrosinase inhibitory peptides derived from food proteins: an overview. Molecules.

[ref-52] Sudjaroen Y, Thongkao K, Saranontawat K, Thongmuang P (2021). Evaluation of anti-oxidant activity and cytotoxicity of aqueous extract from nipa palm (*Nypa fruticans* Wurmb.) vinegar. Natural Volatiles and Essential Oils.

[ref-53] Tayier N, Qin NY, Zhao LN, Zeng Y, Wang Y, Hu G, Wang YQ (2021). Theoretical exploring of a molecular mechanism for melanin inhibitory activity of calycosin in zebrafish. Molecules.

[ref-54] Tian S, Wang J, Li Y, Li D, Xu L, Hou T (2015). The application of in silico drug-likeness predictions in pharmaceutical research. Advanced Drug Delivery Reviews.

[ref-55] Videira IF, Moura DF, Magina S (2013). Mechanisms regulating melanogenesis. Anais Brasileiros de Dermatologia.

[ref-56] Yu Q, Fan L, Duan Z (2019). Five individual polyphenols as tyrosinase inhibitors: Inhibitory activity, synergistic effect, action mechanism, and molecular docking. Food Chemistry.

[ref-57] Yusoff NA, Ahmad M, Hindi BAl, Widyawati T, Yam MF, Mahmud R, Razak KN, Asmawi MZ (2015a). Aqueous extract of *Nypa fruticans* Wurmb, vinegar alleviates postprandial hyperglycemia in normoglycemic rats. Nutrients.

[ref-58] Yusoff NA, Yam MF, Beh HK, Abdul Razak KN, Widyawati T, Mahmud R, Ahmad M, Asmawi MZ (2015b). Antidiabetic and antioxidant activities of Nypa fruticans Wurmb, vinegar sample from Malaysia. Asian Pacific Journal of Tropical Medicine.

[ref-59] Zhang Y, Cai P, Cheng G, Zhang Y (2022). A brief review of phenolic compounds identified from plants: their extraction, analysis, and biological activity. Natural Product Communications.

[ref-60] Zolghadri S, Bahrami A, Hassan Khan MT, Munoz-Munoz J, Garcia-Molina F, Garcia-Canovas F, Saboury AA (2019). A comprehensive review on tyrosinase inhibitors. Journal of Enzyme Inhibition and Medicinal Chemistry.

