# Supplementary material for: Antioxidant activity, anti-tyrosinase activity, molecular docking studies, and molecular dynamic simulation of active compounds found in nipa palm vinegar
Source: PeerJ. 2023 Nov 24;11:e16494. doi: 10.7717/peerj.16494 (PMC10680452; doi:10.7717/peerj.16494)
Supplement: Supplemental Information 1 [file peerj-11-16494-s001.docx]

**Table S1.** Chemo-informatics assessment of natural products from AE O-NPV, NPV-P and AE NPV-P predicted by SwissADME server.

| **Compound Name** | **M: Wt**  **≤ 500** | **HBA**  **≤ 10** | **HBD**  **≤ 5** | **LogP**  **≤ 5** | **PSA**  **≤ 140 (A^2^)** | **Bioavailability Score > 0** | **RO5** |
| --- | --- | --- | --- | --- | --- | --- | --- |
| Vanillic acid | 168.15 | 4 | 2 | 1.08 | 66.76 | 0.85 | Yes |
| Catechin | 290.727 | 6 | 5 | 0.422 | 110.38 | 0.55 | Yes |
| Rutin | 610.50 | 16 | 10 | -1.29 | 266.00 | 0.17 | No |
| Quercetin | 302.24 | 7 | 5 | 0.349 | 127.00 | 0.55 | Yes |
| **Control inhibitors** |  |  |  |  |  |  |  |
| Aloin | 418.40 | 9 | 7 | -0.14 | 167.91 | 0.55 | Yes |
| Deoxyarbutin | 194.23 | 3 | 1 | 2.40 | 38.69 | 0.55 | Yes |
| Arbutin | 272.253 | 7 | 5 | -0.81 | 119.61 | 0.55 | Yes |
| Kojic acid | 142.11 | 4 | 2 | -0.90 | 66.80 | 0.55 | Yes |
| Hexylresorcinol | 194.27 | 2 | 2 | 3.50 | 40.46 | 0.55 | Yes |

M.Wt: molecular weight; HBA: number of hydrogen bond acceptor; HBD: number of hydrogen bond donor, LogP: lipophilicity; LogS: water solubility; PSA: total polar surface area; RO5: Lipinski’s rule of 5.
