# Supplementary material for: Antioxidant activity, anti-tyrosinase activity, molecular docking studies, and molecular dynamic simulation of active compounds found in nipa palm vinegar
Source: PeerJ. 2023 Nov 24;11:e16494. doi: 10.7717/peerj.16494 (PMC10680452; doi:10.7717/peerj.16494)
Supplement: Supplemental Information 2 [file peerj-11-16494-s002.docx]

**Table S2.**  Structure-activity relationship (SAR) of the most potent compounds.

| **Compound Name** | ***In silico* anti-tyrosinase effect** | ***In vitro* anti-tyrosinase effect** | **References** |
| --- | --- | --- | --- |
| Vanillic acid | K_i_ values of 428.75 nM | IC_50_ values of 15.84 mM | Girawale, et al. 2022 |
| Deoxyarbutin | K_i_ values of 37.61 µM | IC_50_ values of 57.70 µM | Chawla et al. 2008 |
| Kojic acid | K_i_ values of 573.31 µM | IC_50_ values of 40.69 µM | Chen et al. 2015 |
