# Supplementary figures and images for: Antioxidant activity, anti-tyrosinase activity, molecular docking studies, and molecular dynamic simulation of active compounds found in nipa palm vinegar
Source: PeerJ. 2023 Nov 24;11:e16494. doi: 10.7717/peerj.16494 (PMC10680452; doi:10.7717/peerj.16494)

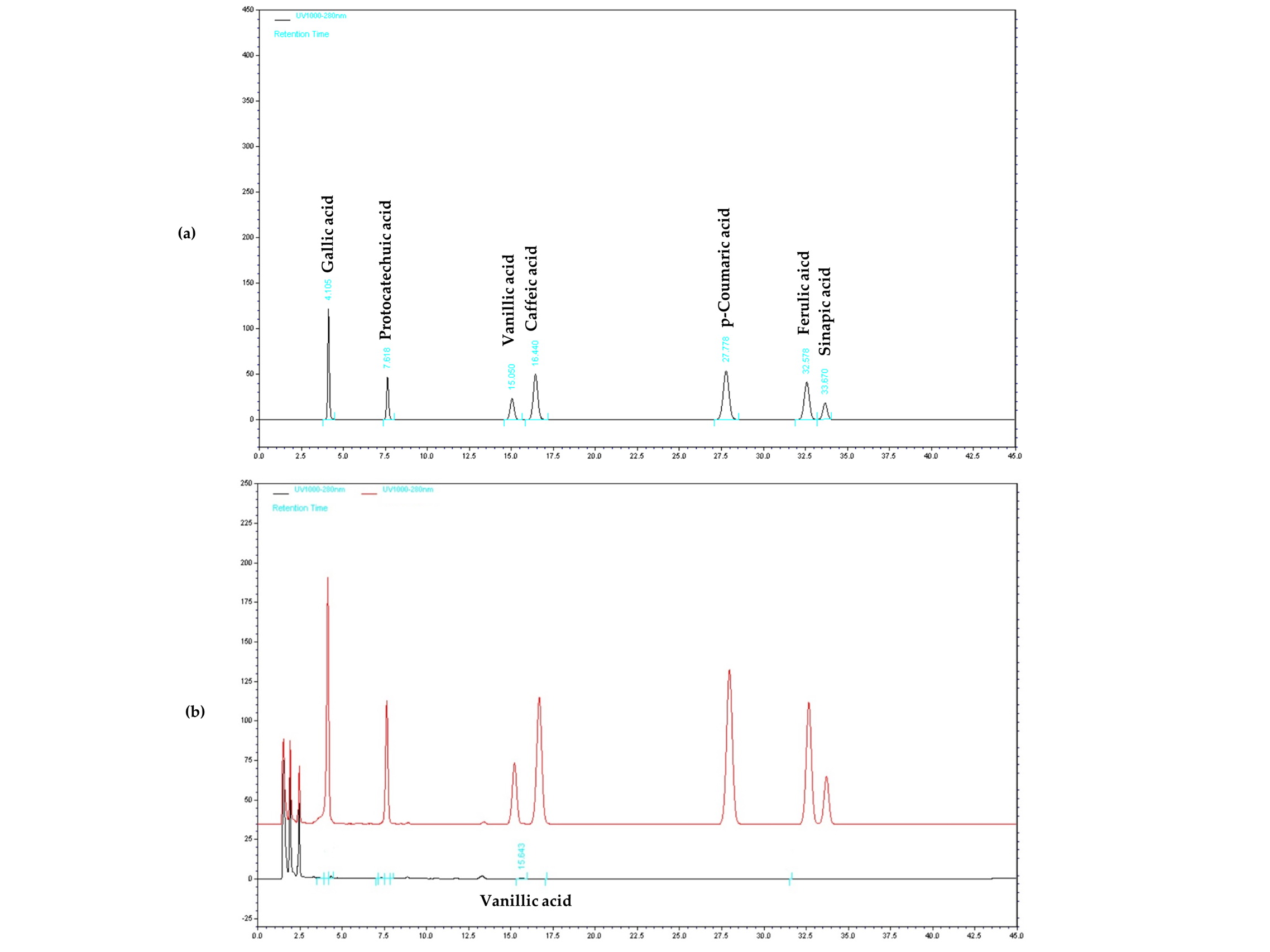

Supplement: Supplemental Information 3 [file peerj-11-16494-s003.jpg]

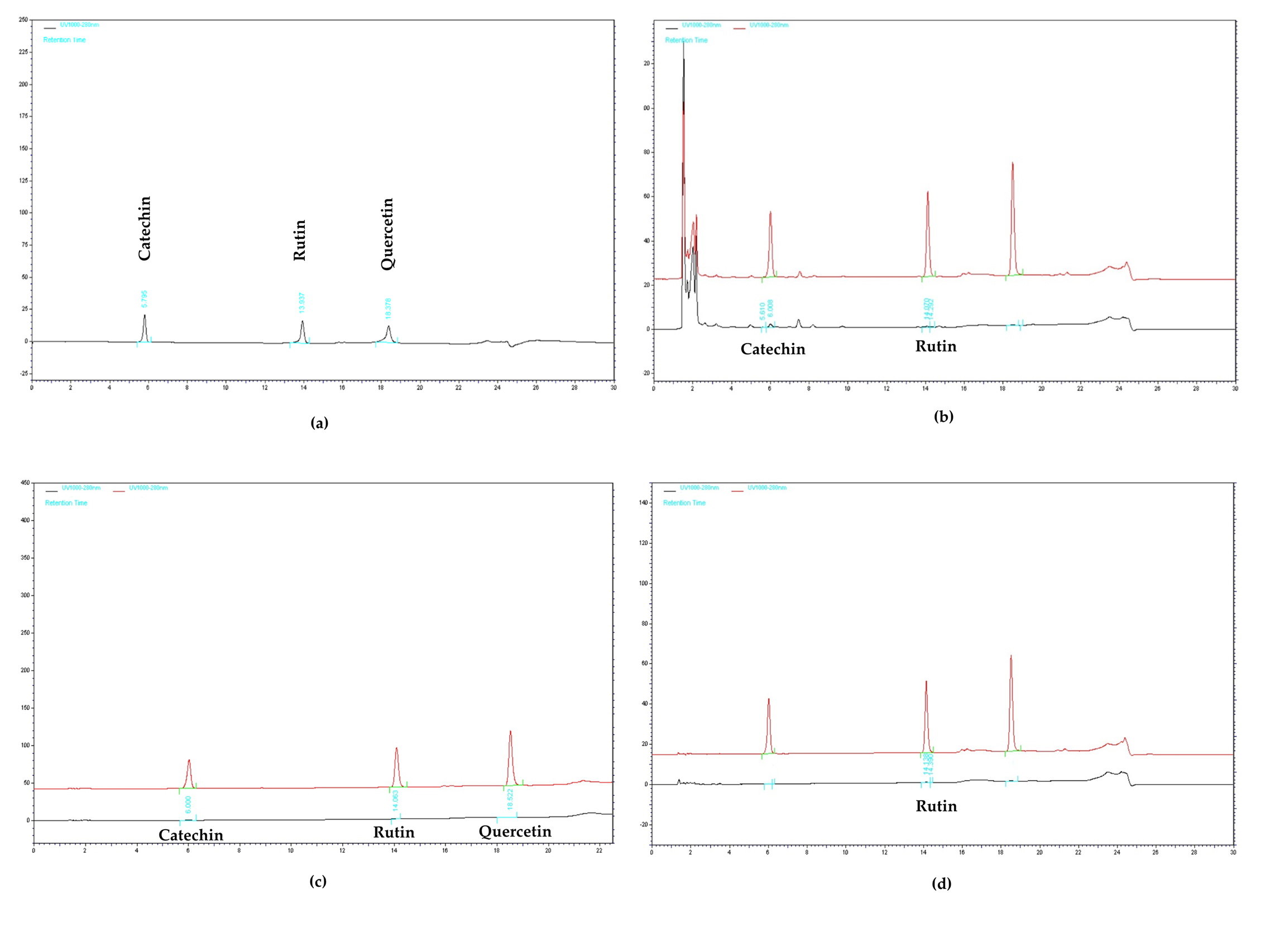

Supplement: Supplemental Information 4 [file peerj-11-16494-s004.jpg]
